# Modulation of Atlantic salmon (*Salmo salar*) gut microbiota composition and predicted metabolic capacity by feeding diets with processed black soldier fly (*Hermetia illucens*) larvae meals and fractions

**DOI:** 10.1186/s42523-021-00161-w

**Published:** 2022-01-15

**Authors:** Pabodha Weththasinghe, Sérgio D. C. Rocha, Ove Øyås, Leidy Lagos, Jon Ø. Hansen, Liv T. Mydland, Margareth Øverland

**Affiliations:** 1 Department of Animal and Aquacultural Sciences, Faculty of Biosciences, Norwegian University of Life Sciences, P.O. Box 5003, 1432 Ås, Norway; 2 Faculty of Chemistry, Biotechnology and Food Science, Norwegian University of Life Sciences, Ås, Norway

**Keywords:** Black soldier fly, Atlantic salmon, Gut microbiota, Predicted microbial metabolic capacity, Full-fat insect meal, Defatted insect meal, De-chitinized insect meal, Insect oil, Insect exoskeleton

## Abstract

**Background:**

Black soldier fly (*Hermetia illucens*) is a promising insect species to use as a novel ingredient in fish feeds. Black soldier fly larvae consists of three major fractions, namely protein, lipid, and exoskeleton. These fractions contain bioactive compounds that can modulate the gut microbiota in fish such as antimicrobial peptides, lauric acid, and chitin. However, it is not certain how, or which fractions of black solider fly would affect gut microbiota in fish. In the present study, black soldier fly larvae were processed into three different meals (full-fat, defatted and de-chitinized) and two fractions (oil and exoskeleton), and included in diets for Atlantic salmon (*Salmo salar*). Atlantic salmon pre-smolts were fed with these diets in comparison with a commercial-like control diet for eight weeks to investigate the effects of insect meals and fractions on the composition and predicted metabolic capacity of gut microbiota. The gut microbiota was profiled by 16S rRNA gene sequencing, and the predicted metabolic capacities of gut microbiota were determined using genome-scale metabolic models.

**Results:**

The inclusion of insect meals and fractions decreased abundance of *Proteobacteria* and increased abundance of *Firmicutes* in salmon gut. The diets that contained insect chitin, i.e., insect meals or exoskeleton diets, increased abundance of chitinolytic bacteria including lactic acid bacteria and *Actinomyces* in salmon gut, with fish fed full-fat meal diet showing the highest abundances. The diets that contained insect lipids, i.e., insect meals and oil diets enriched *Bacillaceae* in fish gut. The fish fed diets containing full-fat insect meal had a unique gut microbiota composition dominated by beneficial lactic acid bacteria and *Actinomyces,* and showed a predicted increase in mucin degradation compared to the other diets.

**Conclusions:**

The present results showed that the dietary inclusion of insect meals and fractions can differently modulate the composition and predicted metabolic capacity of gut microbiota in Atlantic salmon pre-smolts. The use of full-fat black soldier fly larvae meal in diets for salmon is more favorable for beneficial modulation of gut microbiota than larvae processed by separation of lipid or exoskeleton fractions.

**Supplementary Information:**

The online version contains supplementary material available at 10.1186/s42523-021-00161-w.

## Background

Aquaculture has been the fastest growing food production sector over the last three decades and is expected to contribute significantly to the global animal-derived protein budget [1]. There is, however, a major constraint in supply of sustainable ingredients for fish feeds [2]. Fishmeal and fish oil have conventionally been used in fish feeds, but this practice is no longer sustainable due to depletion of wild forage fish, high market prices, conflicts about resource use, and environmental issues [3]. Alternative plant ingredients, such as soy products also raise serious ethical and sustainability concerns related with human food consumption [4], intensified crop production, deforestation, and other environmental issues [5, 6]. The presence of anti-nutritional factors further limits the use of plant ingredients [7]. Hence, aquaculture requires sustainable novel feed ingredients to remain economically and environmentally sustainable.

Over the last few years, there has been a growing interest in using insects as a sustainable novel fish feed ingredient [8]. Although the production volumes of insects cannot yet compete with conventional feed sources [8], the approval for use of processed insects in aqua feed by the European Commission (Regulation 2017/893/EC, 2017) promotes upscaling of insects as a fish feed ingredient. Due to the high nutritive value [9], low environmental impacts [10–12] and suitability for large scale production [13], black soldier fly (*Hermetia illucens*) (BSF) becomes a promising insect species to use for feed purposes. During the past decade, an increasing number of studies have successfully used BSF in diets for different fish species including Atlantic salmon (*Salmo salar*). The majority of studies showed that BSF did not compromise growth performance in salmon at low to moderate dietary inclusion levels [14–16], while other studies also showed positive effects of feeding BSF on gut health of salmon [17], confirming its potential as a novel ingredient in salmon feeds.

The gut microbiota plays a crucial role in digestive function, nutrient metabolism, growth performance, fish physiology, barriers against pathogens, immune response, disease resistance, welfare, and health in fish [18–23]. Thus, a beneficial gut microbiota can be a key factor to improve nutrient utilization, growth performance, and health in fish. Diet is one of the main drivers in shaping the gut microbiota [24, 25]. Feeding diets containing BSF meal was previously reported to modulate gut microbiota in salmon [26] and rainbow trout (*Oncorhynchus mykiss*) [27–29]. The BSF consists of three major fractions namely protein, lipid, and exoskeleton [30]. Each fraction contains different bioactive compounds, such as antimicrobial peptides (AMP), lauric acid and chitin, respectively [31]. The expanded spectrum of AMP present in BSF have activity against many bacteria [32–35], while lauric acid has demonstrated antimicrobial effects against Gram-positive bacteria [36–38]. Dietary chitin has shown antimicrobial and bacteriostatic activity against several Gram-negative pathogens [39] but also to enrich beneficial microbiota in salmon gut due to its prebiotic properties [28, 29]. Hence, it is possible that the BSF might modulate gut microbiota, which in turn could affect fish nutrient utilization, growth, and health. However, it is not certain how, or which specific compounds in BSF would affect gut microbiota in salmon. Characterizing the response of salmon gut microbiota to dietary full-fat BSF meal compared with different fractions of BSF and further processed BSF meals by separating lipid or exoskeleton fraction is, thus, worthy of attention. It is further important to determine how BSF should be processed to optimize its use in salmon diets. Although previous studies used full-fat and defatted BSF meals in salmonid diets, to the best of our knowledge, no studies evaluated the effects of different meals and fractions of BSF larvae on gut microbiota in a single study.

To date, majority of studies on gut microbiota of fish fed BSF have been restricted to analysis of taxonomic composition. Few previous studies showed that insect-based feeds could modulate the functional repertoire of gut microbiota in fish [40, 41] and the functional alterations of the gut microbiota to dietary insects varied with the fish species [40]. Nevertheless, we are still far from understanding how BSF and its specific compounds affect the functional profile of gut microbiota in Atlantic salmon, which is essential to identify potential fish-microbiota interactions. Therefore, the aims of the present study were to compare the composition, diversity and predicted metabolic capacities of gut microbiota in Atlantic salmon pre-smolts when fed with BSF larvae meals (full-fat, defatted and de-chitinized meals) and fractions (oil and exoskeleton) by high-throughput sequencing technology.

## Methods

### Experimental diets, fish study and sampling

The BSF larvae were reared and processed into three meals (full-fat, defatted and de-chitinized) and two fractions (oil and exoskeleton) at HiProMine S.A., Robakowo, Poland. The BSF larvae were dried at 110 °C for 1 h and then at 80 °C for 23 h until a constant weight was reached using a chamber air flow dryer (HiProMine S.A., Robakowo, Poland) to produce full-fat BSF larvae meal. A part of dried full-fat meal was defatted using oil press (Reinartz, model AP14/22, Neuss, Germany) to produce defatted meal and oil. The larvae were mechanically de-chitinized using food press twin-screw processor with 0.3 mm screen diameter (Angel Juicer, model 7500, Busan, Korea), and dried at 110 °C for 1 h and then at 80 °C for 23 h until a constant weight was reached using a chamber air flow dryer (HiProMine S.A., Robakowo, Poland) to produce de-chitinized meal and exoskeleton fraction. Additional file 2: Table S1 shows the composition of BSF meals and fractions. Further details on rearing of BSF larvae and composition of BSF meals and fractions have been reported in [42]. The fatty acid (FA) content of BSF larvae lipid fraction was determined using Trace GC Ultra gas chromatograph (Thermo Fisher Scientific, US) according to O'fallon et al. [43]. The FA composition of the lipid fraction of BSF larvae is shown in Additional file 2: Table S2. The BSF larvae were rich in saturated FA (71% of total FAs), mainly lauric acid (40% of total FAs). Six experimental diets were formulated to meet NRC [44] nutrient requirements of Atlantic salmon; a commercial-like control diet containing fishmeal, plant protein meals and fish oil (CD); three diets containing BSF meals and two diets containing BSF fractions. The three BSF meal diets contained either full-fat (IM), defatted (DFIM) or de-chitinized (DCIM) BSF meal replacing 15% of the protein content of CD. Two BSF fractions diets contained either BSF oil (IO) or exoskeleton (EX). The oil and exoskeleton of BSF were added to the diets to match the BSF oil and chitin contents in IM diet, respectively. Table 1 shows the ingredient and chemical compositions of the six experimental diets. The feed was produced with extrusion technology using a five-section Bühler twin-screw extruder (BCTG 62/20 D, Uzwil, Switzerland) without a pre-conditioner. After the extrusion, the pellets were vacuum coated with fish oil and/or BSF larvae oil in Gentle Vacuum Coater (GVC)—80 prototype (Fôrtek, Amandus-Kahl). Further details on the feed processing in the present study have been reported in [42].

**Table 1:** Ingredient and chemical composition of experimental diets containing meals or fractions of black soldier fly (BSF) larvae^a^

	CD	IM	DFIM	DCIM	IO	EX
*Ingredients (%)*						
Fishmeal	22.50	18.57	18.57	18.57	22.50	21.78
Soy protein concentrate	34.50	28.48	28.48	28.48	34.50	33.39
Corn gluten	5.50	4.54	4.54	4.54	5.50	5.32
Full-fat BSF larvae meal	0.00	20.36	0.00	0.00	0.00	0.00
Defatted BSF larvae meal	0.00	0.00	14.89	0.00	0.00	0.00
De-chitinized BSF larvae meal	0.00	0.00	0.00	24.53	0.00	0.00
BSF larvae oil	0.00	0.00	0.00	0.00	6.24	0.00
BSF larvae exoskeleton	0.00	0.00	0.00	0.00	0.00	7.20
Wheat flour	14.65	14.65	14.65	14.65	14.65	14.65
Fish oil	16.00	10.47	14.75	5.82	10.05	15.36
Methionine	0.20	0.20	0.20	0.20	0.20	0.20
Choline chloride	0.15	0.15	0.15	0.15	0.15	0.15
Yttrium	0.01	0.01	0.01	0.01	0.01	0.01
Vit/min premix	0.65	0.65	0.65	0.65	0.65	0.65
Monocalcium Phosphate	0.80	0.80	0.80	0.80	0.80	0.80
Wheat bran	5.04	1.12	2.31	1.60	4.75	0.49
*Chemical composition (%, wet-weight basis)*						
Dry matter	91.6	91.9	93.0	92.9	93.3	91.7
Crude protein	46.6	44.4	46.0	46.6	46.6	47.3
Crude lipid	19.6	20.3	17.8	12.9	18.3	17.0
Starch	13.1	12.2	12.4	12.4	12.6	11.7
Ash	6.70	6.60	6.77	7.23	6.70	6.61
Chitin		1.44	1.44	0.53		1.43

^a^*CD* Control diet; *IM* Full-fat BSF larvae meal diet; *DFIM* Defatted BSF larvae meal diet; *DCIM* De-chitinized BSF larvae meal diet; *IO* BSF larvae oil diet; *EX* BSF larvae exoskeleton diet

The fish study was conducted at the Center for Fish Research, NMBU, Ås, Norway. A total of 900 Atlantic salmon pre-smolts (Aqua Gen Atlantic QLT-innOva SHIELD) (28 g of average initial weight) were allocated into 18 fiberglass tanks, and fed ad libitum with one of the six experimental diets (n = 3) for eight weeks. The fish were reared in recirculated freshwater (14.4 ± 0.4 °C) and kept under continuous light. At the end of the experiment, six fish from each tank were randomly sampled and anesthetized using tricaine methanesulfonate (MS-222) (80 mg/L). Fish were euthanized by a sharp blow to the head and recorded individual weight. The distal intestine was defined as the darker color section of the intestine with large diameter and annular rings [45]. The distal intestine was opened longitudinally and, the digesta was removed carefully using sterile plastic spatulas. The digesta was placed in cryotubes, snap-frozen in liquid nitrogen and stored at − 80 °C. To maintain aseptic conditions, the digesta samples were collected near a gas burner and tools were cleaned and decontaminated using 70% ethanol spray and flaming between each fish. In addition, feed samples and water samples from the tank water source were collected into sterile plastic containers and stored at − 80 °C.

### Extraction of DNA from samples and controls

Total DNA from approximately 200 mg of digesta (18 samples per dietary group) and 100 mg of ground feed (2 samples per diet) was extracted using QIAamp Fast DNA Stool Mini Kit (Qiagen, Hilden, Germany, Cat. No. 51604) according to the guidelines of the manufacturer with the following modifications: For the lysis of the samples, 300 µL (for digesta samples) or 500 µL (for feed samples) of Buffer ASL (Stool lysis buffer, Cat No./ID: 19082) were added to 2 mL prefilled bead tubes (Qiagen; Cat No., 13118-50) (100 µL of 0.1 mm glass beads). Then the samples were homogenized in a bead mill homogenizer (Qiagen RETSCH tissuelyser) at 20 Hz twice for 3 min, with a pause of 2 min (on ice) between the runs. The temperature for the heating incubation was 95 °C, and after adding proteinase K and buffer AL the incubation was 15 min at 90 °C. The extracted DNA was eluted with 50 μl of Buffer ATE and incubated 10 min at room temperature before centrifugation. In addition to digesta and feed, total DNA was extracted from two water samples. Water samples (500 mL) were filtered through a MF-Millipore membrane filter with 0.22 µm pore size (Sigma-Aldrich, Cat No. GSWP04700) and DNA was extracted using the same method as above but 600 μl of buffer ASL was added for the lysis.

To assess the reliability of the present workflow, two controls were added during DNA extraction: a blank negative control without a sample and a positive control containing a microbial community standard (mock), which consists of eight bacteria and two yeasts (Zymo- BIOMICS™, Zymo Research, California, USA; catalog no., D6300). The same DNA extraction procedure used for digesta samples was followed for both negative and positive (75 µL) controls. Further, the total DNA was extracted from a blank filter paper used for the filtration of water following the same procedure used for DNA extraction from water. After extraction, the DNA concentration was determined in duplicates using Invitrogen™ Quant-iT™ Qubit™ dsDNA HS (High sensitivity) assay kit (Thermo Fisher Scientific, California, USA, Cat No: Q32854) with the Qubit 4 Fluorometer (Invitrogen). The extracted DNA were stored at − 20 °C until further analysis.

### PCR amplification

A first PCR (in duplicates) was performed in 25 μL reactions to amplify the V3–V4 hypervariable regions of the bacterial 16S rRNA gene. The primers used were 341F (5’-CCTACGGGNGGCWGCAG-3’) and 785R (5’-GACTACHVGGGTATCTAATCC-3’). The reaction mix contained 2 × KAPA HiFi HotStart Ready Mix (12.5 μL) (Roche Sequencing Solutions, Material No: 7958935001), DNA template (5 μL), and 1.33 μM primers (3.75 μL of each primer). The PCR thermal cycling began with an initial denaturation at 95 °C for 3 min and followed by 30 cycles of denaturation at 95 °C for 30 s, annealing at 55 °C for 30 s, and extension at 72 °C for 30 s, and a final extension at 72 °C for 5 min. The duplicate amplified PCR products were pooled and purified using Agencourt AmPure XP beads (Beckman Coulter, Indiana, USA, Cat No: A63881). The cleaned PCR products were examined by 1% agarose gel electrophoresis. The 13 digesta samples with strongest bands of each dietary group were used for sequencing.

### Library preparation and sequencing

The library preparation was conducted according to the Illumina 16S Metagenomic Sequencing Library Preparation protocol [46]. First, the cleaned PCR amplicons were indexed with the Nextera XT Index Kit v2 Set A (96 indexes, 384 samples) (Illumina, California, USA, Cat. No: FC-131-2001) in eight PCR cycles. The index PCR products were purified using the AMPure beads and quantified using the Invitrogen™ Quant-iT™ Qubit™ dsDNA BR (Broad Range) assay kit (Thermo Fisher Scientific, California, USA, Cat no: Q32853) with the Qubit 4 Fluorometer (Invitrogen). The library size was determined using representative cleaned libraries with the Agilent DNA 1000 Kit (Agilent Technologies, California, USA; catalog no., 5067–1505). The libraries were diluted to 4 nM in 10 mM Tris (pH 8.5). The libraries from negative control and blank filter paper had a concentration lower than 4 nM, and thus were not diluted. Equal volumes of diluted and undiluted libraries were pooled. The pooled library was denatured using 0.2 N NaOH. A standard Illumina generated PhiX control (Illumina, San Diego, Waltham, MA, USA, Cat No: FC-110–3001) was also denatured. The denatured library was combined with 5% Phix control (570 μL library + 30 μL PhiX control). The combined library and Phix control was then loaded at 8 pM and sequenced on the Miseq System (Illumina, San Diego, California, USA) using the Miseq Reagent Kit v3 (600-cycle) (Illumina; catalog no., MS-102-3003). The clustering density was 804 K/mm2 and 91% of clusters were passing filter. Data output from the sequencer were demultiplexed FASTQ format files.

### Processing of sequence data

The processing of sequence data was done in R 4.0.4 [47]. The DADA2 1.18.0 was used to process the raw sequence data and generate amplicon sequence variants (ASVs) [48]. A total of 15.7 million raw reads were generated for digesta, feed, and water samples. The median of raw reads per sample were 165,648, while the minimum reads per sample was 46,075 and maximum was 815,888. The median Phred quality score of reads was crashed at position 298 bp in forward reads and at position 220 bp in reverse reads. The primer sequences and low-quality reads from where the median Phred quality score crashed were trimmed and filtered out from the demultiplexed paired ended reads. A model of error rates was developed, and error sequences were removed. The forward and reverse reads from each sample were merged (with 36 bp overlap), ASV table was constructed, and chimeric sequences were removed from the ASV table. A total number of 14,666 unique ASVs were generated after the sequence denoising and ASVs filtering for chimeric sequences. The resulted ASVs were assigned with taxonomy using the reference database, Silva version 138.1 [49, 50]. The sequences obtained from the mock samples were matched with the expected reference sequences to evaluate the DADA2 performance. A phyloseq object was built using the R package phyloseq 1.34.0 using the generated ASV table, taxonomy table and sample metadata [51]. The undetermined sample in the sequence output was removed from the phyloseq object. The following ASVs were removed from the ASV table: ASVs identified as chloroplasts (4.7% of ASVs) or mitochondria (10% of ASVs), ASVs with no phylum-level taxonomic assignment and ASVs found in only one non-negative control sample. The contaminating ASVs due to reagent contamination and cross contamination were identified and removed from ASV table as explained by Li et al. [26]. The resulted ASV table contained 3590 unique ASVs. The ASVs were then clustered using VSEARCH algorithm and subsequently curated with LULU [52]. This post-clustering curation reduced the number of unique ASVs to 2660. The resulted ASV table was used for further analyses. Taxonomic analysis showed that 69.4% of ASVs were assigned at the genus level whereas only 10.6% of ASVs had a species-level annotation. The core ASVs, alpha diversity indices (observed ASVs, Pielou’s evenness, Shannon’s index and Faith’s phylogenetic diversity (PD)) and beta diversity indices (Jaccard distance, unweighted UniFrac distance, Aitchison distance and PHILR transformed Euclidean distance) were computed according to Li et al. [26]. The ASV table was rarefied based on minimum sequence size (10,332) in the samples to compute Jaccard distance and unweighted UniFrac distance (Fig. S1).

### Metabolic reaction level analysis

The reaction-level analysis of gut microbiota was performed as previously described by Yilmaz et al. [53]. The ASVs were mapped to metabolic reactions via an available collection of genome-scale metabolic models (GSMMs) of gut microbes [54], including only ASVs that could be mapped to a taxonomic rank of family or lower and to at least one GSMM. For each sample, we then calculated the normalized abundance of each reaction (i):

$$a_{r} \left( i \right) = \frac{{\mathop \sum \nolimits_{j = 1}^{n} a_{{{\text{ASV}}}} \left( j \right)E\left( {i,j} \right)}}{{\mathop \sum \nolimits_{j = 1}^{n} a_{{{\text{ASV}}}} \left( j \right)}}$$

where $a_{{{\text{ASV}}}} \left( j \right)$ is the abundance of ASV *j* in the sample, *n* is the total number of ASVs, and $E\left( {i,j} \right)$ is the expected probability (frequency of occurrences) of reaction *i* in the GSMMs mapped to ASV *j*.

### Statistical analysis

Linear discriminant analysis (LDA) effect size (LEfSe) tool [55] was used to characterize microbial differences of biological relevance between the dietary groups. The statistical differences were evaluated using factorial Kruskal–Wallis rank sum test, followed by pairwise Wilcoxon test and a threshold between 3.5 and 4.0 for the LDA. The strategy for multi-class analysis was one-against-all/all-against-all. The statistical analyses related to microbial diversity were run in R 4.1.0 [47]. The statistical difference between the dietary groups for the four alpha diversity indices were evaluated using Kruskal–Wallis test, followed by multiple comparisons using Wilcox pair-wise comparison test. The differences in beta-diversity were evaluated by performing permutation multivariate analysis of variance (PERMANOVA) [56] with 999 permutations using the R package vegan 2.5.7 [57], and followed by a pair-wise comparison. The four beta-diversity distance matrices were visualized by the principal coordinates analysis (PCoA). The homogeneity of multivariate dispersions among groups was evaluated by the permutation test, PERMDISP [58], using the R package vegan 2.5.7 [57]. The adjusted pair-wise comparisons by the Benjamini- Hochberg procedure were used where applicable [59]. Differences were regarded as significant when *p* < 0.05.

All analyses related to metabolic reactions were performed in Python 3.7.0. A two-sample *t*-test was used to compare the mean abundances of each metabolic reaction for each pair of diets. The Benjamini–Hochberg procedure [59] was used to correct for multiple testing, and reactions with adjusted *p* ≤ 0.05 were considered to be significantly different between diets. The metabolic pathway classification of reactions was obtained from the GSMMs, and Fisher’s exact test was used to identify enriched pathways among the significantly different reactions. The pathways with Benjamini-Hochberg-adjusted *p* ≤ 0.05 were considered to be enriched. Further, principal component analysis (PCA) was performed separately on standardized ASVs (Additional file 1: Fig. S2) and reaction abundances (z-scores) (Additional file 1: Fig. S3).

## Results

### Microbiota associated with positive and negative controls

Confirming the reliability of the present workflow for assessing the gut microbiota, the eight expected bacterial genera in the mock were successfully identified. *Staphylococcus aureus* was identified at the species level as well (Additional file 1: Fig. S4). The relative abundance of *Enterococcus*, *Listeria* and *Staphylococcus* were underestimated, whereas the relative abundance of *Bacillus*, *Lactobacillus* and *Pseudomonas* were overestimated. The Pearson correlation coefficient (Pearson’s *r*) between the expected and observed taxonomic profile of the mock at genus level was 0.48, while it was 0.99 between the observed profiles. The dominant taxa identified in the blank filter paper were *Paracoccus* (21%) and *Corynebacterium* (22%). The contaminant taxa of the negative control was dominated by *Candidatus Nomurabacteria* (23%).

### Microbiota associated with water and feed

**Fig. 1:**
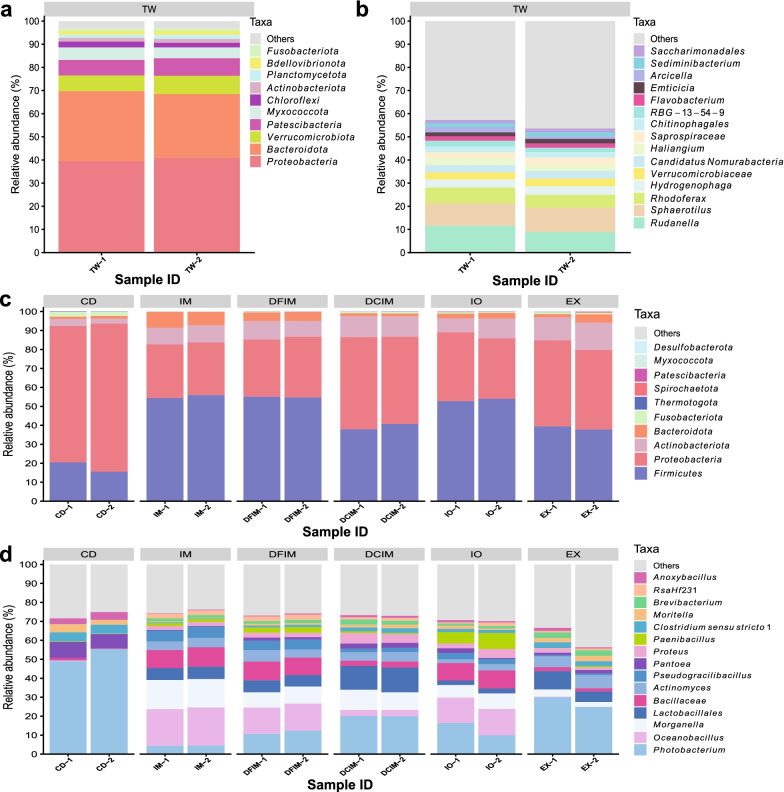
Most abundant taxa in tank water (TW) and feed samples. Top 10 most abundant taxa at phylum (comprised 97% of abundance) (**a**) and top 15 most abundant taxa at genus or lowest taxonomy level (comprised 55% of abundance) (**b**) in water samples. Top 10 most abundant taxa at phylum (comprised 100% of abundance) (**c**) and top 15 most abundant taxa at genus or lowest taxonomy level (comprised 61–75% of abundance) (**d**) in feed samples. The plots display the relative taxa abundances in all the feed and water samples. The feed samples are grouped by the diet. *CD* control diet; *IM* full-fat BSF (black soldier fly) larvae meal diet; *DFIM* defatted BSF larvae meal diet; *DCIM* de-chitinized BSF larvae meal diet; *IO* BSF larvae oil diet; *EX* BSF larvae exoskeleton diet

The microbiota in tank water were dominated by phyla *Proteobacteria* (40%), *Bacteroidota* (29%), *Verrucomicrobiota* (7%), and *Patescibacteria* (7%) (Fig. 1a). At the genus or lowest taxonomy level, *Rudanella* (10%), *Sphaerotilus* (10%), *Rhodoferax* (6%), *Hydrogenophaga* (4%) and *Verrucomicrobiaceae* (3%) dominated the microbiota in tank water (Fig. 1b).

The taxonomic compositions of the feed samples were diet-dependent (Fig. 1c, d and Additional file 1: Fig. S5). At the phylum level, the microbiota in the feed was dominated, regardless of the diet, by *Proteobacteria*, *Firmicutes* and *Actinobacteriota*. The CD feed had higher abundance of *Proteobacteria* (75%) compared to insect-based feed (28–47%). On the contrary, insect-based feed had higher abundance of *Firmicutes* (18% in CD and 39–55% in insect-based feed), and *Actinobacteriota* (3% in CD and 9–13% in insect-based feed) (Fig. 1c and Additional file 1: Fig. S5a). At genus or lowest taxonomic level, microbiota associated with insect-based feed showed higher abundance of *Oceanobacillus*, *Actinomyces*, *Brevibacterium*, *Lactobacillales*, *Bacillaceae*, *Pseudogracilibacillus* and *RsaHf*231 compared to CD feed, while *Morganella* was solely found in insect-based feed pellets. The CD feed was dominated by *Photobacterium* (52%) (Fig. 1d and Additional file 1: Fig. S5b).

### Gut-associated microbiota

**Fig. 2:**
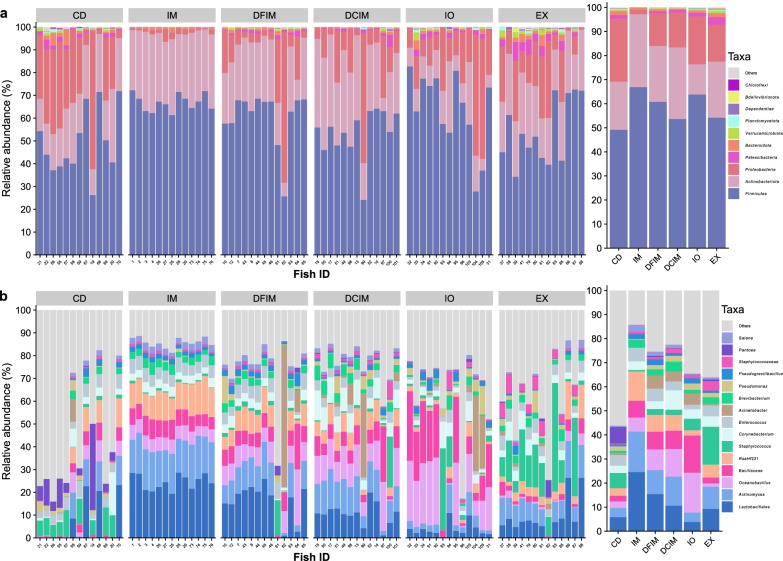
Most abundant taxa in distal intestine digesta samples from fish fed experimental diets. Top 10 most abundant taxa at phylum (comprised 99.6–100% of abundance) (**a**) and top 15 most abundant taxa at genus or lowest taxonomy level (comprised 44–86% of abundance) (**b**). The plots in left side of the figure display the relative taxa abundances in all the samples. The samples are grouped by the diet. The plots in right side display the mean abundance of each taxon within the same dietary group. *CD* control diet; *IM* full-fat BSF (black soldier fly) larvae meal diet; *DFIM* defatted BSF larvae meal diet; *DCIM* de-chitinized BSF larvae meal diet; *IO* BSF larvae oil diet; *EX* BSF larvae exoskeleton diet

The taxonomic composition of the digesta samples were diet-dependent (Fig. 2). At the phylum level, the gut microbiota of fish fed insect-based diets had higher abundance of *Firmicutes* (54–67%) and lower abundance of *Proteobacteria* (2–20%) than the fish fed CD (49% and 26% respectively). The fish fed insect diets except IO also had higher abundance of *Actinobacteriota* (20% in CD and 23–30% in insect-based groups) (Fig. 2a). At genus or lowest taxonomic level, *Lactobacillales* was the dominant taxon in IM (25%) and DFIM (15%) groups. Insect meals and EX groups had higher abundance of *Actinomyces* (9–17%) compared to the CD and IO groups (4%). The insect diets fed fish except EX showed higher abundance of *Bacillaceae* (7–15%), compared to CD diet fed fish (2%). The DCIM group had the highest abundance of *Corynebacterium* (8%). The IO and EX groups were dominated by *Oceanobacillus* (17%) and *Staphylococcus* (16%) respectively, whereas *Pantoea* (7%) and *Staphylococcus* (6%) were dominant in CD group (Fig. 2b).

**Fig. 3:**
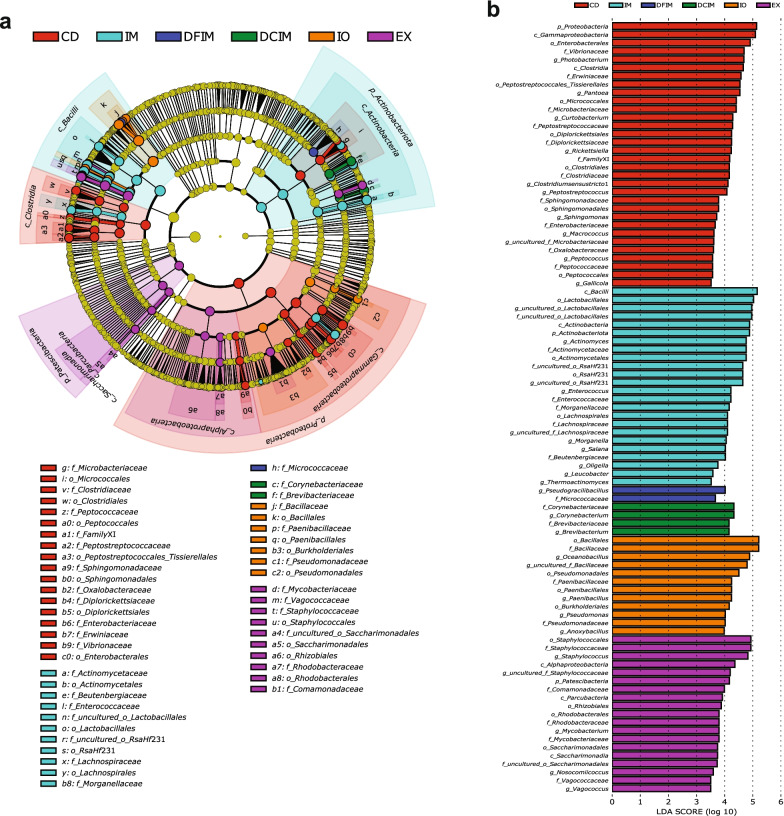
Linear discriminant analysis (LDA) effect size (LEfSe) results on gut microbiota of fish. Circular cladogram reporting LEfSe results presents the identified amplicon sequence variants (ASVs) distributed according to phylogenetic characteristics around the circle (**a**). The dots closest to the center represent ASVs at the phylum level, whereas the outer circle of dots represent ASVs at the genus level. The color of the dots and sectors indicate the dietary group in which the respective ASVs are most abundant. The color explanation is given above the cladogram. Yellow color indicates ASVs that showed similar abundance in all dietary groups. The colored sectors give information on phylum, class (full name in outermost circles, given only for phylum or class showing significant difference between groups), order, family, and genus (indicated by letter). The explanation is given below the cladogram. Indicator taxa with LDA scores of 3.5 or greater in the microbial communities (**b**). *p* phylum; *c* class; *o* order; *f* family; *g* genus; *CD* control diet; *IM* full-fat BSF (black soldier fly) larvae meal diet; *DFIM* defatted BSF larvae meal diet; *DCIM* de-chitinized BSF larvae meal diet; *IO* BSF larvae oil diet; *EX* BSF larvae exoskeleton diet

To characterize the microbiota in fish gut with significant differences in abundances between the dietary groups, LEfSe was performed. The LEfSe results are presented in cladograms showing the phylogenetic distribution of the bacterial lineages and LDA column charts. Figure 3 shows the significantly enriched taxa in all the dietary groups. At LDA score of 3.5, most of the significantly enriched taxa in CD group belonged to classes *Gammaproteobacteria* and *Clostridia*, while *Photobacterium* and *Vibrionaceae* were among the enriched taxa in *Gammaproteobacteria*. The significantly enriched taxa in IM group mainly belonged to phylum *Actinobacteriota* and class *Bacilli*, such as *Lactobacillales*, *Enterococcaceae*, *RsaHf*231, *Actinomyces* and *Enterococcus*. The DFIM significantly enriched family *Micrococcaceae* and genus *Pseudogracilibacillus*, whereas DCIM group had significantly higher abundance of genera *Corynebacterium* and *Brevibacterium*. The IO mainly enriched order *Bacillales*, and genera *Oceanobacillus*, *Paenibacillus*, *Anoxybacillus* and *Pseudomonas*. The EX mainly enriched phylum *Patescibacteria* and genera *Staphylococcus* and *Mycobacterium*.

**Fig. 4:**
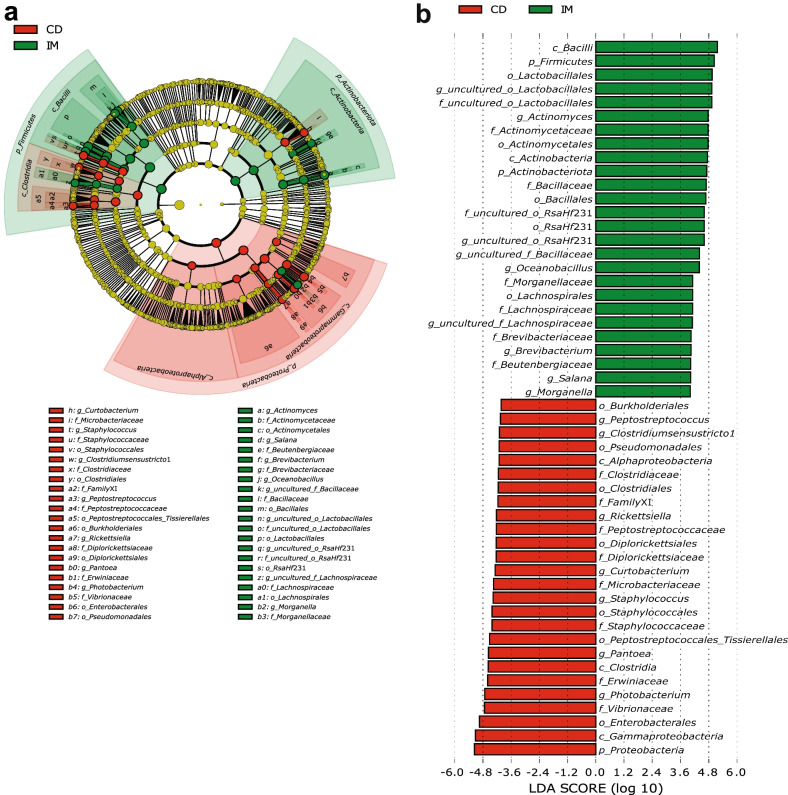
Linear discriminant analysis (LDA) effect size (LEfSe) results on gut microbiota of fish fed CD and IM diets. Circular cladogram reporting LEfSe results presents the identified amplicon sequence variants (ASVs) distributed according to phylogenetic characteristics around the circle (**a**). The dots closest to the center represent ASVs at the phylum level, whereas the outer circle of dots represent ASVs at the genus level. The color of the dots and sectors indicate the dietary group in which the respective ASVs are most abundant. The color explanation is given in the upper left corner. Yellow color indicates ASVs that showed similar abundance in all dietary groups. The colored sectors give information on phylum, class (full name in outermost circles, given only for phylum or class showing significant difference between groups), order, family, and genus (indicated by letter). The explanation is given below the cladogram. Indicator taxa with LDA scores of 4 or greater in the microbial communities (**b**). *p* phylum; *c* class; *o* order; *f* family; *g* genus; *CD* control diet; *IM* full-fat black soldier fly larvae meal diet

Figure 4 shows the LEfSe results for the comparison between CD and IM groups at LDA score of 4. LEfSe detected 52 bacterial clades (26 in each) showing statistically significant different abundances between the IM and CD groups. In comparison with CD group, IM diet enriched taxa belong to two main classes namely *Actinobacteria* and *Bacilli*. The enriched taxa in these classes included *RsaHf*231, *Lactobacillales*, *Bacillales*, *Bacillaceae*, *Actinomyces*, *Oceanobacillus*, and *Brevibacterium*. Most of these bacterial taxa were also significantly enriched in DFIM, DCIM and EX groups compared to CD group (Additional file 1: Fig. S6-8). In addition, DCIM diet also enriched genera *Acinetobacter* and *Corynebacterium* (Additional file 1: Fig. S7), and EX group enriched *Staphylococcus* compared to CD (Additional file 1: Fig. S8). The EX group, however, did not enrich *Bacillaceae* and *Oceanobacillus* (Additional file 1: Fig. S8). The IO diet mainly enriched taxa belonging to class *Bacilli* such as *Bacillaceae* and *Oceanobacillus* compared to the CD group (Additional file 1: Fig. S9).

The gut microbial compositions in the gut partly resembled the microbiota in respective feed, but differed from the water microbiota. The ASVs overlap between the gut and feed was higher than that between the gut and water (Additional file 1: Fig. 5).

**Fig. 5:**
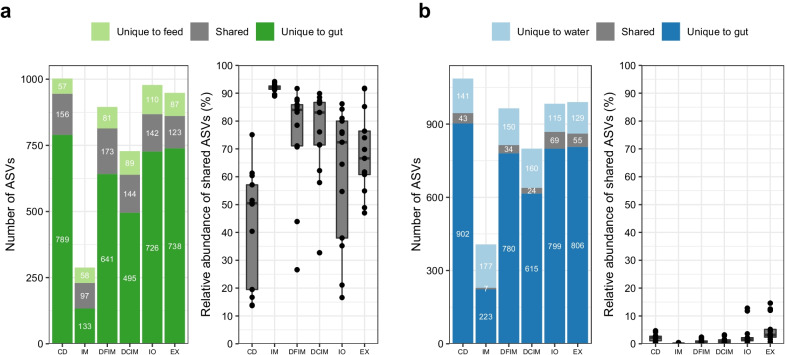
The microbial overlap between the gut and feeds (**a**) and between gut and water (**b**). The number of shared amplicon sequence variants (ASVs) is shown on the left side in each panel. The relative abundance of shared ASVs is shown on the right side in each panel. The minimum relative abundance of ASVs to be considered as present in a sample was 0.05%. *CD* control diet; *IM* full-fat BSF (black soldier fly) larvae meal diet; *DFIM* defatted BSF larvae meal diet; *DCIM* de-chitinized BSF larvae meal diet; *IO* BSF larvae oil diet; *EX* BSF larvae exoskeleton diet

At prevalence threshold of 80%, 173 ASVs were identified as core microbiota in fish gut. Two ASVs, classified as *Enterococcus* and *Lactobacillales* were identified as core ASVs in all the digesta sample types (Additional file 3: Table S3). Additionally, fish fed insect diets shared 13 ASVs identified as *RsaHf*231, *Oceanobacillus caeni*, *Actinomyces*, *Corynebacterium urealyticum*, *Staphylococcaceae*, *Lactobacillales*, *Pseudogracilibacillus* and *Brevibacterium* (Additional file 1: Fig. S10a; Additional file 3: Table S3). The insect meal groups (IM, DFIM and DCIM) had 46 core ASVs and *Atopostipes, Globicatella, Companilactobacillus*, *Enterococcus*, *Corynebacterium*, *Brevibacterium senegalense*, *Oceanobacillus* and *Morganella* were among them (Additional file 1: Fig. S10b; Additional file 3: Table S3). The four diets that contained BSF lipid (IM, DFIM, DCIM and IO) shared 26 ASVs and most of them belonged to family *Bacillaceae* (Additional file 1: Fig. S10c; Additional file 3: Table S3). The four diets which contained BSF chitin (IM, DFIM, DCIM and EX) had 17 shared ASVs and *Enterococcus*, *Vagococcus*, and *Corynebacterium* were among them (Additional file 1: Fig. S10d; Additional file 3: Table S3).

### Alpha diversity

**Fig. 6:**
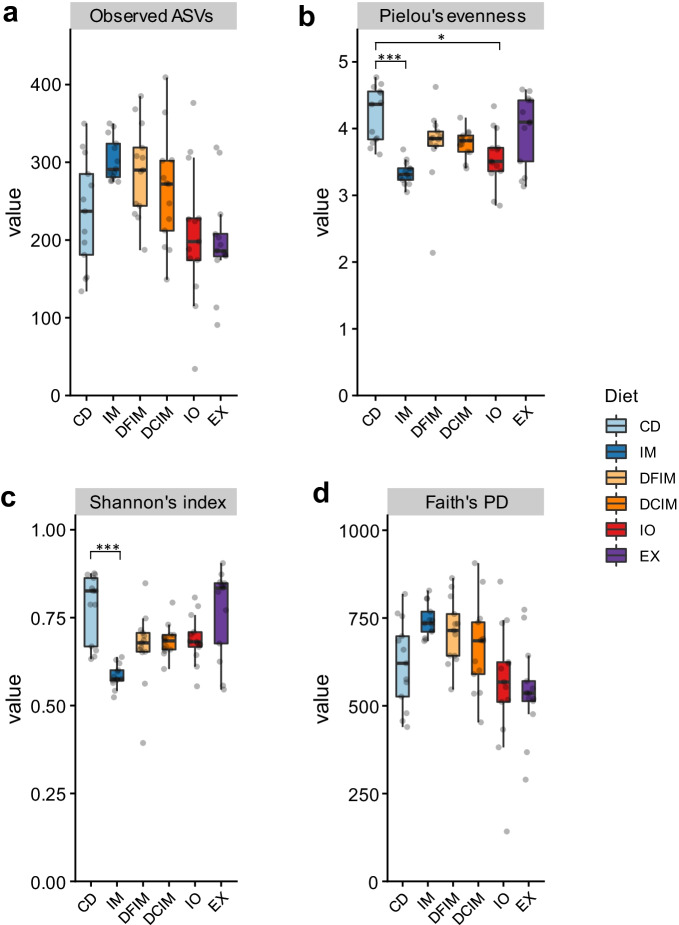
The alpha-diversity of gut microbiota in salmon fed experimental diets. Observed Amplicon sequence variants (ASVs) (**a**). Pielou's evenness (**b**). Shannon's index (**c**). Faith's phylogenetic diveristy (PD) (**d**). Asterisks denote statistically significant differences between control group and insect groups (*p < 0.05; ***p < 0.001). *CD* control diet; *IM* full-fat BSF larvae meal diet; *DFIM* defatted BSF larvae meal diet; *DCIM* de-chitinized BSF (black soldier fly) larvae meal diet; *IO* BSF larvae oil diet; *EX* BSF larvae exoskeleton diet

The diet significantly affected the alpha diversity indices of gut microbiota (*p* < 0.001) (Fig. 6 and Additional file 2: Table S4). The observed ASVs did not differ between the gut microbiota in insect groups and CD group, but IM group presented a numerically higher average (Fig. 6a). The IM and IO groups showed lower Pielou’s evenness than the CD group, and the other groups did not differ from CD group (Fig. 6b). Further, IM diet also reduced Shannon’s index compared to CD group (Fig. 6c). Following the trend for observed ASVs, IM group had numerically higher average Faith’s PD compared to the CD group (Fig. 6d). The Shannon’s index and Faith’s PD in other groups did not differ from CD group (Fig. 6c, d). Additionally, the observed ASVs and Faith’s PD were higher in IM group than IO and EX groups, but did not differ from DFIM and DCIM groups. Pielou’s evenness was lower in IM compared to DFIM and DCIM groups, while Shannon’s index in IM group was lower than other insect groups (Additional file 2: Table S4). Hence, the fish fed IM diet seems to have a different gut microbial composition dominated by specific bacterial group(s) compared to those fed CD and other insect-based diets.

### Beta diversity

**Fig. 7:**
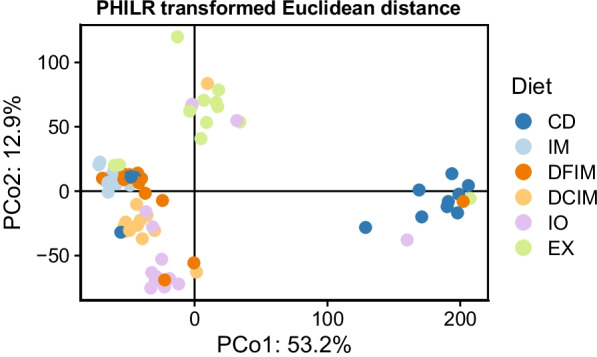
The beta-diversity (based on phylogenetic isometric log-ratio (PHILR) transformed Euclidean distance matrix) of gut microbiota in fish fed experimental diets. *PCo* principal coordinate; *CD* control diet; *IM* full-fat BSF (black soldier fly) larvae meal diet; *DFIM* defatted BSF larvae meal diet; *DCIM* de-chitinized BSF larvae meal diet; *IO* BSF larvae oil diet; *EX* BSF larvae exoskeleton diet

The PCoA plots for all four beta-diversity indices showed that insect groups separated from CD group (Fig. 7 and Additional file 1: Fig. S11). Confirming the group separation in PCoA plots, PERMANOVA results also revealed differences between the gut microbiota of fish fed CD and insect diets in at least one of the distance matrices used. Although it was not clear in the PCoA plots, the statistical tests showed differences in beta-diversity between microbiota in the IM group and the other insect groups, regardless of the distance matrix used (Additional file 2: Table S5). The box-plots and results of the tests for homogeneity of multivariate dispersions are shown in Additional file 1: Fig. S12 and Additional file 2: Table S6, respectively. For the four distances, IM and DCIM groups showed lower multivariate dispersions than the CD group, whereas no differences were observed between CD group and DFIM and EX groups. In addition, the IM diet had the lowest multivariate dispersions among the insect groups for all the four distances.

### Metabolic capacity of gut microbiota

Of the 2660 ASVs, 1374 could be mapped to at least one genome-scale metabolic model (GSMM) from a published collection of GSMMs of gut microbes [54]. Among these, 868 were matched to family with an average of 16 models per ASV, 464 were matched to genus with an average of 10 models per ASV, and 42 were matched to species with an average of 1 model per ASV (Additional file 1: Fig. S13). In total, the models that were mapped to ASVs contained 4886 different reactions. Most of these reactions (78%) were present in all samples and all samples contained at least 82% of the reactions, but the abundances of many reactions differed significantly between samples and diets. Furthermore, PCA of reaction abundances allowed much more of the variability in the data to be explained in a few components than PCA of ASV abundances (Additional file 1: Figs. S2 and S3).

**Fig. 8:**
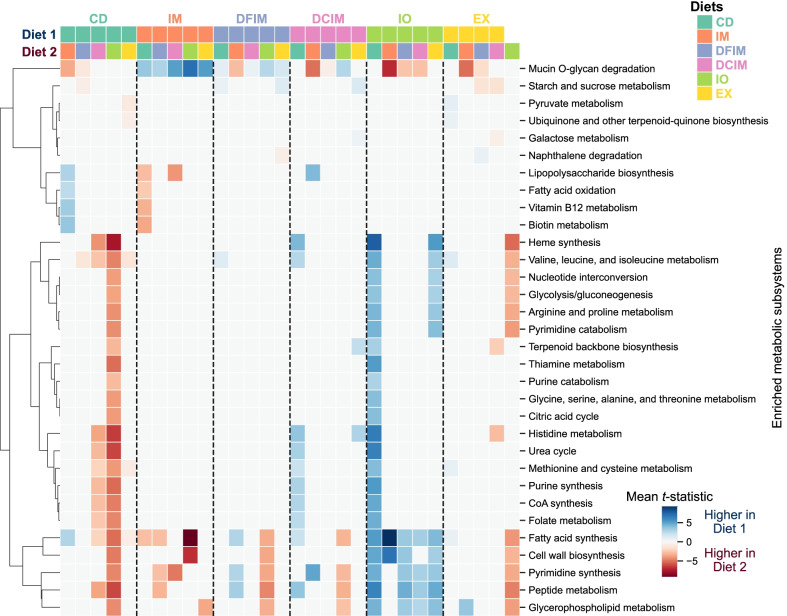
Hierarchical clustering of the significantly enriched metabolic subsystems between each pair of dietary groups. Columns are diet pairs, rows are metabolic subsystem, and the color of each cell indicates whether the metabolic subsystem was enriched in diet 1 (blue) or diet 2 (red). *CD* control diet; *IM* full-fat BSF (black soldier fly) larvae meal diet; *DFIM* defatted BSF larvae meal diet; *DCIM* de-chitinized BSF larvae meal diet; *IO* BSF larvae oil diet; *EX* BSF larvae exoskeleton diet

Grouping reactions by metabolic pathways, we found that 32 pathways were enriched in reactions with significantly different mean abundances between dietary groups (Fig. 8). The mean differences in reaction abundances between groups are shown for enriched pathways in Additional file 1: Fig. S14. The gut microbiota in fish fed IO indicated the highest number of enriched pathways (22) compared to the CD group (Fig. 8; Additional file 1: Fig. S14a). The first principal component of PCA on reaction abundances (*z*-scores) showed that IM and IO groups separated from the other groups in terms of their predicted metabolic capacities (Additional file 1: Fig. S3c). These two groups showed predicted enrichment of metabolic pathways mucin O-glycan degradation and FA synthesis, respectively, compared to other groups (Fig. 8).

In comparison with CD group, the IM group was predicted to decrease lipopolysaccharide biosynthesis, vitamin metabolism and FA synthesis and oxidation (Fig. 8; Additional file 1: Fig. S14d), whereas DFIM group showed a predicted enrichment of mucin O-glycan degradation, starch and sucrose metabolism and valine, leucine, and isoleucine metabolism (Fig. 8; Additional file 1: Fig. S14i). The DCIM, IO and EX showed a predicted enrichment of metabolic pathways related to amino acid metabolism and FA synthesis compared to the CD group (Fig. 8; Additional file 1: Fig. S14a, c, h).

## Discussion

### Modulation of fish gut microbiota composition and diversity

The present study showed that inclusion of meals and fractions of insects in the diet can modify the gut microbiota of Atlantic salmon pre-smolts. Previous findings also showed that feeding BSF modulated gut microbiota in rainbow trout [27–29, 41, 60] and salmon post-smolts [26]. The phyla *Firmicutes*, *Proteobacteria*, *Actinobacteriota*, and *Bacteroidota* represented more than 94% of the gut microbiota in fish, regardless of the diet, which belong to the core gut microbiota in different fish species [22]. The observed increase of *Firmicutes*, *Actinobacteriota*, *Lactobacillales*, *Actinomyces*, *RSaHf231*, *Oceanobacillus*, *Bacillaceae*, *Brevibacterium, Acinetobacter*, *Staphylococcus* and/or *Corynebacterium* and decreased *Proteobacteria* abundances in comparison to the control fish, also observed previously in gut microbiota of salmon post-smolts [26] and rainbow trout [27, 29] when fed BSF meal. Although *Mycoplasma* has been identified as a core taxon in salmon in several studies [61–63], this was absent in the gut microbiota of fish in the present study. Similar results were observed in salmon pre-smolts reared in a freshwater recirculating aquaculture system [64]. The presence and abundance of *Mycoplasma* may be attributed to early life exposure to *Mycoplasma* [64] and rearing environment [65].

The observed gut microbiota differed from water-associated microbiota. This can be related to the low water intake of fish in freshwater, and is in accordance with previous studies showing that gut microbiota of rainbow trout and salmon reared in freshwater did not reflect the microbiota of the surrounding environment [21, 66]. The highly abundant taxa in feed containing insect meals and fractions were *Oceanobacillus*, *Actinomyces, Brevibacterium*, *Lactobacillales*, *Bacillaceae*, *RsaHF*231, and *Morganella*. Such taxa have also been identified in BSF whole larvae/prepupae or their gut [67–70]. The microbiota in all diets contained *Photobacterium*, but made up more than 50% of the microbiota in the control feed, similar to previous reports [60]. *Photobacterium* is widely distributed in marine environment and fish [71, 72]. Thus, it is plausible that fishmeal made from marine fish was the main source of *Photobacterium* in the feed in the present study and its abundance is associated with the fishmeal inclusion level. In future studies, analyzing the microbiota in the feed ingredients would provide useful information regarding the sources of microbes in the feed.

The modulation of gut microbiota in fish fed the insect-based diets can be explained by microbiota associated in feed and the composition of feed. There were overlaps between the microbes found in feed and fish gut, in particular the bacterial taxa, *Pantoea*, *Oceanobacillus*, *Lactobacillales*, *Bacillaceae, Actinomyces* and *RsaHf*231. High overlap between the microbiota associated with gut and feed has also been observed previously in salmon pre-smolts fed insect meal [66]. While high temperature during extrusion could eliminate microbiota in feed, dead bacteria and spores can still be profiled by the DNA sequencing technique. Hence, the observed microbial composition in the fish gut could reflect some dead or inactivated microbes in undigested feed. However, it is also possible that resistant bacterial spores could modulate microbial community in the gut, but the extent to which the observed feed microbiota contributed to shape gut microbiota cannot be identified using sequencing-based methods. For instance, feed and gut associated microbiota shared family *Bacillaceae* in several dietary groups in the present study. The family *Bacillaceae* consists *Bacillus* which forms spores resistant to extrusion processing [73, 74]. Our results also suggested that fish gut microbiota was not merely originated from feed, but that the specific feed composition modulated the microbiome.

Insect meals and exoskeleton fraction enriched *Lactobacillales*, which are commonly known as lactic acid bacteria (LAB). Among the dietary groups, the full-fat group had the highest abundance of *Lactobacillales* and *Enterococcus*, a genus belonging to *Lactobacillales*. Previous studies also showed that dietary inclusion of BSF meal increased abundance of *Lactobacillales*, *Lactobacillus* and/or *Enterococcus* in salmon post-smolts [26] and rainbow trout [27, 29, 41]. The LAB are commonly observed microbes of the teleost fish gut in minor proportions of the overall community [75, 76]. A recent study also showed metagenomic assembled genomes of both *Enterobacteriaceae* and *Lactobacillus* in the gut of rainbow trout [77]. In general, LAB are considered as beneficial gut microbes due to their abilities to enhance digestive function, mucosal tolerance, immune response, and disease resistance in host [78]. They are known to produce lactic acid and bactericidal compounds that may prevent colonization of pathogens on the intestinal surface [78–80] and even repair or prevent the intestinal damage caused by antinutritional factors present in plant-based ingredients such as soybean meal in fish [81].

The exoskeleton of BSF contains chitin [82], which can be associated with proliferation of LAB due to its prebiotic properties [18, 28, 29]. Our results strongly supported this, since only the diets containing insect chitin (1.4% in full-fat meal, defatted meal, and exoskeleton diets and 0.5% in de-chitinized meal diet) enriched abundance of *Lactobacillales* in the fish gut. In addition to LAB, these four chitin containing diets also increased abundance of *Actinomyces* in gut microbiota in fish, with the highest abundance observed from full-fat meal diet. The enrichment of *Actinomyces* has previously been shown when salmon post-smolts [26] and rainbow trout [29, 60] were fed BSF meal. *Actinomyces* are often identified as chitin degraders and might benefit from the presence of chitin [83]. The genus *Actinomyces* is within the class *Actinobacteria*, which is involved in the function of the intestinal barrier of the fish and playing an essential role in the synthesis of antimicrobial compounds against fish pathogens [84]. In addition, many bacterial species belonging to *Bacillus* of family *Bacillaceae* can produce chitinase [85, 86]. In the present study, the insect meals and oil diets enriched *Bacillaceae* in the fish gut. Huyben et al. [27] also showed similar results in rainbow trout fed full-fat or defatted BSF larvae meals. Hence, in the present study, chitin in the BSF larvae could have acted as a substrate and may have selectively promoted the growth of certain chitinolytic bacteria in the fish gut such as *Lactobacillales*, *Actinomyces* and *Bacillaceae* in agreement with previous observations in Atlantic salmon [85] and Atlantic cod (*Gadus morhua* L.) [87].

The lipid fraction of BSF larvae was rich with medium chain lauric acid (40% of total FAs), and contained negligible level of long-chain polyunsaturated omega-3 FAs (Additional file 2: Table S2). This FA composition of BSF can also be responsible partially for increased LAB abundance as shown by Rimoldi et al. [80] and Huyben et al. [88], although the insect oil diet did not enrich LAB in the present study. Fish fed de-chitinized meal showed the highest abundance of *Corynebacterium* in gut microbiota as observed in rainbow trout fed BSF larvae or pre-pupae meal [27, 29]. During the de-chitinization process, there was an increase of the relative lipid content of insect meal (44%) (Additional file 2: Table S1), making de-chitinized meal diet the one with the highest level of BSF lipids. Thus, BSF lipids might cause the increase in *Corynebacterium* in the gut of fish fed de-chitinized meal diet. Huyben et al. [27] also observed that the abundance in fish fed full-fat BSF meals were higher than in fish fed defatted meal. The *Corynebacterium* has been reported to produce lipase [89]. The de-chitinized meal decreased abnormal lipid accumulation in the enterocytes of pyloric caeca (P. Weththasinghe, J.Ø. Hansen, L.T. Mydland, L. Lagos, B. Morales-Lange, M. Øverland, unpublished observations), and it is possible that the enriched *Corynebacterium* might have played a role in preventing this condition. Moreover, despite of being chitinolytic bacteria, *Bacillaceae* were only enriched in fish fed BSF lipid containing diets and not in exoskeleton diet with insect chitin, indicating BSF lipid fraction was favorable for the proliferation of this bacteria. In the present study, the exoskeleton diet gave the highest abundance of *Staphylococcus*, followed by the control diet. The other insect diets, which contained lauric acid, did not enrich *Staphylococcus*. Lauric acid has shown antimicrobial activity against some species of *Staphylococcus* [90]. This suggested that BSF lipid can also modulate salmon gut microbiota in addition to chitin. Altogether, BSF as a whole or its components, might explain the changes in the microbial community, and most importantly, the reduced abundance of Gram-negative *Gammaproteobacteria*, *Vibrionaceae* and *Photobacterium* in fish fed insect-based diets in comparison to control fish. Chitin was previously reported to have antimicrobial and bacteriostatic activity against several Gram-negative pathogens [39]. Furthermore, Rimoldi et al. [80] reported that lauric acid in the diet can also reduce the abundance of *Gammaproteobacteria*. The observed decrease in *Proteobacteria* abundance and increase in *Firmicutes* abundance in insect-based groups can be beneficial. *Proteobacteria,* especially *Gammaproteobacteria* comprehends several potentially pathogenic bacterial species for fish [91] and phylum *Firmicutes* contains beneficial microbes for fish [29].

In the present study, fish fed full-fat meal diet presented a numerically higher average of species richness, as shown by observed ASVs. The decreased Pielou’s evenness suggested that the fish fed full-fat meal and insect oil might have specific bacterial group(s) that dominated the gut microbiota, also supported by Shannon’s index of full-fat meal group. Faith’s PD measures the biodiversity, based on the phylogeny distance, showed that phylogenetic diversity in fish fed full-fat meal was higher (at least tended to) than that in fish fed other diets, indicating the tendency for the presence of species from diverse clades in the phylogeny tree. Hence the alpha-diversity indices strongly indicated that fish fed full-fat meal might have a different gut microbial composition from those fed other diets, and there might be a dominance of a specific group(s), i.e., LAB and *Actinomyces*. Higher abundance of these chitinolytic bacteria can be the reason for increased phylogenetic diversity in full-fat meal group, since a higher phylogenetic diversity exists within chitinolytic bacteria [83]. On the contrary to the results for fish fed full-fat insect meal, the species richness, evenness, and the diversity in gut microbiota in fish fed defatted and de-chitinized meals did not differ from the control fish. Previous studies, however, showed that feeding defatted BSF meal increased richness and diversity in rainbow trout [29, 41] and salmon post-smolts [26]. The beta diversity indices showed that gut microbiota of insect-based groups separated from that of the control group, as previously observed in rainbow trout [27–29, 41]. Regardless of the distance matrix used, gut microbiota in full-fat meal group also differ from the other insect groups. High gut microbial diversity can be associated with positive health effects. Species-rich communities are thought to potentially provide further metabolic capabilities to the host [92] and out-compete pathogens for nutrients and colonization [93, 94], e.g., LAB can reduce pathogens adhesion by creating a biofilm in the gut [80, 95]. Nevertheless, increased bacterial diversity can also indicate dysbiosis of the gut microbiota [96], which is generally associated with reduced performance of fish.

The growth performances of fish in the present study have been reported elsewhere [42]. The fish fed full-fat meal showed higher growth performance compared to the fish fed control diet and other insect-based diets. The unique gut microbiota composition in full-fat group, together with the enrichment and dominance of beneficial bacteria such as LAB and *Actinomyces*, may cause this improvement in growth. It is possible that chitin, lauric acid and other bioactive components such as AMP might have acted together for beneficial modulation of gut microbiota in fish fed full-fat meal, and consequently improved fish growth performance. Altogether, this points to the use of full-fat BSF larvae meal in diets for Atlantic salmon as more efficient, than processed larvae by separation of lipid or exoskeleton fractions.

### Modulation of metabolic capacity of gut microbiota

The gut microbiota carries out many metabolic reactions, which play a critical role in host nutrition, physiological functions, and health [41, 97]. In the present study, we used a metagenome prediction tool to predict the metabolic capacity of gut microbiota of fish. However, the reliability of such prediction tools is questionable due to the biased databases towards human-related microbiota [98]. In particular, the GSMM collection used in the present study was originally created for microbes found in human gut microbiota. Furthermore, we could only use the ASVs that match to known GSMM, which did not represent the whole microbiota in fish gut. Considering these limitations, the predicted metabolic profiles should be interpreted with caution, and metatransciptomic, metaproteomic or metabolomic analyses of digesta samples would be preferred to determine the real functional profile of gut microbiota. Meta-omics methods such as high-resolution untargeted metabolomics, metatranscriptomics and metaproteomics combined with metagenomics have successfully been used in other studies to profile the functions of gut microbiota in fish [77, 99, 100]. However, the predicted metabolic pathways can still provide an indication of metabolic capacity of gut microbiota and such prediction tools have widely been used in studies to determine the modulation of function repertoire of gut microbiota of fish in response to the diet. For instance, Parris et al. [100] reported consistent results for functional enrichments of gut microbiota in clownfish (*Premnas biaculeatus*) when using metagenome prediction and metatranscriptomics.

In the present study, the predicted metabolic reaction profile of gut microbiota in fish fed full-fat BSF meal diet differed from other diets, as observed in PCA results. The full-fat insect meal enriched mucin O-glycan degradation in gut microbiota compared to the control as well as other insect-based diets. The mucus layer covering intestinal epithelium is mainly consisted of mucin with a vast array of O-glycan structures [101]. Mucus nature can benefit certain mucin-degrading bacteria and thereby, shaping the gut microbiota composition at the mucosal surface, gut inflammatory responses [102] and host immune responses [103]. The gut microbiota of fish fed full-fat insect meal was dominated by LAB which contains species with mucin binding protein and are capable of adhering to the intestinal mucin [104, 105]. Nevertheless, previous studies have shown that LAB isolated from aquatic animals cannot degrade porcine mucin in vitro [106, 107]. This strengthens the importance of validation of these metabolic changes using meta-omics techniques. At the same time, it was also observed lower levels of reactions in lipopolysaccharide biosynthesis pathway in fish fed full-fat meal diet compared to fish fed control and de-chitinized meal diets. Gram-negative bacteria produce and have lipopolysaccharides on cell surface [108, 109], which are recognized as pathogen-associated molecules and can activate the innate immune response in fish [110]. This reduction in lipopolysaccharide biosynthesis is in accordance with drastic reduction in Gram-negative *Proteobacteria* in full-fat meal group. In addition, gut microbiota in fish fed full-fat meal indicated predicted decrease in vitamin metabolism, supporting the previous observations in European seabass (*Dicentrarchus labrax*) and gilthead sea bream (*Sparus aurata*) fed BSF meal [40]. Considering the growth performance of fish in the present study [42], it is likely that full-fat meal benefited the metabolic activity of Atlantic salmon gut microbiota and consequently the fish growth.

The predicted enrichment of FA synthesis in fish fed BSF lipid-rich de-chitinized meal and oil can mostly be due to the lack of omega-3 FAs in lipid fraction of BSF larvae (Additional file 2: Table S2). Gut microbiota can compensate for low levels of omega-3 FAs in the diet by increasing the abundance of FA producing bacteria [88]. In agreement with this, Huyben et al. [88] showed that low omega-3 FAs in a diet containing 23% lipid led to a predicted increase in microbial FA synthesis in salmon. In contrast, the full-fat meal diet did not give a predicted increase in microbial FA synthesis although this diet contained similar BSF lipid content as BSF oil diet. This can be related to the lower abundance of *Proteobacteria* because many of the previously reported bacterial omega-3 producers belong to the class *Gammaproteobacteria* [111]. Feeding defatted meal caused a predicted enrichment of starch and sucrose metabolism in gut microbiota, and similar results were previously shown by Rimoldi et al. [41] and Panteli et al. [40] in rainbow trout and European sea bass, respectively, when fed BSF meal. This indicates gut microbiota of fish fed with defatted insect meal may have the capacity to improve dietary carbohydrates utilization by complementing the endogenous digestive enzymes [41].

## Conclusions

The present results showed that feeding meals and fractions of BSF insect larvae differently modulated gut microbial composition, diversity, and predicted metabolic repertoire in Atlantic salmon pre-smolt. Both insect meals and fractions decreased *Proteobacteria* abundance and increased *Firmicutes* abundance in the gut of fish. The diets containing BSF chitin, i.e., insect meals and exoskeleton diets, increased chitinolytic LAB and *Actinomyces*, while those containing BSF lipids, i.e., insect meals and oil diets, increased the abundance of *Bacillaceae*. Full-fat insect meal led to a unique gut microbiota composition dominated by the beneficial LAB and *Actinomyces*, and showed a predicted increase in mucin degradation compared to the fish fed other diets. Overall, the present results showed that full-fat BSF larvae meal was more favorable for beneficial modulation of gut microbiota than processed larvae by separation of lipid and exoskeleton fractions.

## Supplementary Information

**Additional file 1: Figure S1**. Rarefaction curves based on observed amplicon sequence variants (ASVs) for the different sample types. The ASV table was rarefied based on minimum sequence size (10,332) in the sample for normalization of the sequence for computation of two of the beta-diversity indices (Jaccard distance and unweighted UniFrac distance). CD: control diet; IM: full-fat BSF (black soldier fly) larvae meal diet; DFIM: defatted BSF larvae meal diet; DCIM: de-chitinized BSF larvae meal diet; IO: BSF larvae oil diet; EX: BSF larvae exoskeleton diet. **Figure S2.** Principal component analysis (PCA) on standardized amplicon sequence variants (ASVs). Score plots for PC1 and PC2 (**a**) and PC1 and PC3 (**b**), mean scores (dark) with 95% confidence intervals for PC1 (**c**), PC2 (**d**), and PC3 (**e**), and percentage of variance explained by PCs (**f**). PC: principal component, CD: control diet; IM: full-fat BSF (black soldier fly) larvae meal diet; DFIM: defatted BSF larvae meal diet; DCIM: de-chitinized BSF larvae meal diet; IO: BSF larvae oil diet; EX: BSF larvae exoskeleton diet. **Figure S3.** Principal component analysis (PCA) on metabolic reaction abundances (*z*-scores). Score plots for PC1 and PC2 (**a**) and PC1 and PC3 (**b**), mean scores (dark) with 95% confidence intervals for PC1 (**c**), PC2 (**d**), and PC3 (**e**), and percentage of variance explained by PCs (**f**). PC: principal component, CD: control diet; IM: full-fat BSF (black soldier fly) larvae meal diet; DFIM: defatted BSF larvae meal diet; DCIM: de-chitinized BSF larvae meal diet; IO: BSF larvae oil diet; EX: BSF larvae exoskeleton diet. **Figure S4.** Expected and observed taxonomic profiles of the mock microbial community standard. Mock_1, Mock_2: observed taxonomic profiles of the mock. Mock_Exp: expected taxonomic profile of the mock. **Figure S5.** Most abundant taxa in feed samples. Top 10 most abundant taxa at phylum (comprised 100% of abundance) (**a**) and top 15 most abundant taxa at genus or lowest taxonomy level (comprised 61–75% of abundance) (**b**) in feed samples. The plots display mean abundance of each taxon within the same diet. CD: control diet; IM: full-fat BSF (black soldier fly) larvae meal diet; DFIM: defatted BSF larvae meal diet; DCIM: de-chitinized BSF larvae meal diet; IO: BSF larvae oil diet; EX: BSF larvae exoskeleton diet. **Figure S6.** Linear discriminant analysis (LDA) effect size (LEfSe) results on gut microbiota of fish fed CD and DFIM diets. Circular cladogram reporting LEfSe results presents the identified amplicon sequence variants (ASVs) distributed according to phylogenetic characteristics around the circle (**a**). The dots closest to the center represent ASVs at the phylum level, whereas the outer circle of dots represent ASVs at the genus level. The color of the dots and sectors indicate the dietary group in which the respective ASVs are most abundant. The color explanation is given in the upper left corner. Yellow color indicates ASVs that showed similar abundance in all dietary groups. The colored sectors give information on phylum, class (full name in outermost circles, given only for phylum or class showing significant difference between groups), order, family, and genus (indicated by letter). The explanation is given below the cladogram. Indicator taxa with LDA scores of 4 or greater in the microbial communities (**b**). p: phylum; c: class; o: order; f: family; g: genus; CD: control diet; DFIM: defatted black soldier fly larvae meal diet. **Figure S7.** Linear discriminant analysis (LDA) effect size (LEfSe) results on gut microbiota of fish fed CD and DCIM diets. Circular cladogram reporting LEfSe results presents the identified amplicon sequence variants (ASVs) distributed according to phylogenetic characteristics around the circle (**a**). The dots closest to the center represent ASVs at the phylum level, whereas the outer circle of dots represent ASVs at the genus level. The color of the dots and sectors indicate the dietary group in which the respective ASVs are most abundant. The color explanation is given in the upper left corner. Yellow color indicates ASVs that showed similar abundance in all dietary groups. The colored sectors give information on phylum, class (full name in outermost circles, given only for phylum or class showing significant difference between groups), order, family, and genus (indicated by letter). The explanation is given below the cladogram. Indicator taxa with LDA scores of 4 or greater in the microbial communities (**b**). p: phylum; c: class; o: order; f: family; g: genus; CD: control diet; DCIM: de-chitinized black soldier fly larvae meal diet. **Figure S8.** Linear discriminant analysis (LDA) effect size (LEfSe) results on gut microbiota of fish fed CD and EX diets. Circular cladogram reporting LEfSe results presents the identified amplicon sequence variants (ASVs) distributed according to phylogenetic characteristics around the circle (**a**). The identified ASVs are distributed according to phylogenetic characteristics around the circle. The dots closest to the center represent ASVs at the phylum level, whereas the outer circle of dots represent ASVs at the genus level. The color of the dots and sectors indicate the dietary group in which the respective ASVs are most abundant. The color explanation is given in the upper left corner. Yellow color indicates ASVs that showed similar abundance in all dietary groups. The colored sectors give information on class (full name in outermost circles, given only for phylum or class showing significant difference between groups), order, family, and genus (indicated by letter). The explanation is given below the cladogram. Indicator taxa with LDA scores of 4 or greater in the microbial communities (**b**). c: class; o: order; f: family; g: genus; CD: control diet; EX: black soldier fly larvae exoskeleton diet. **Figure S9.** Linear discriminant analysis (LDA) effect size (LEfSe) results on gut microbiota of fish fed CD and IO diets. Circular cladogram reporting LEfSe results presents the identified amplicon sequence variants (ASVs) distributed according to phylogenetic characteristics around the circle (**a**). The dots closest to the center represent ASVs at the phylum level, whereas the outer circle of dots represent ASVs at the genus level. The color of the dots and sectors indicate the dietary group in which the respective ASVs are most abundant. The color explanation is given in the upper left corner. Yellow color indicates ASVs that showed similar abundance in all dietary groups. The colored sectors give information on phylum, class (full name in outermost circles, given only for phylum or class showing significant difference between groups), order, family, and genus (indicated by letter). The explanation is given below the cladogram. Indicator taxa with LDA scores of 4 or greater in the microbial communities (**b**). p: phylum; c: class; o: order; f: family; g: genus; CD: control diet; IO: black soldier fly larvae oil diet. **Figure S10.** Venn’s diagram showing the shared and unique core ASVs in digesta samples belong to insect-based groups (**a**), insect meal groups (**b**), insect lipid containing groups (**c**) and insect chitin containing groups (**d**). The core ASVs were computed using a prevalence threshold of 80%. CD: control diet; IM: full-fat BSF (black soldier fly) larvae meal diet; DFIM: defatted BSF larvae meal diet; DCIM: de-chitinized BSF larvae meal diet; IO: BSF larvae oil diet; EX: BSF larvae exoskeleton diet. **Figure S11.** The beta-diversity (based on Jaccard, unweighted UniFrac, and Aitchison distance matrices) of gut microbiota in fish fed experimental diets. PCo: principal coordinate; CD: control diet; IM: full-fat BSF (black soldier fly) larvae meal diet; DFIM: defatted BSF larvae meal diet; DCIM: de-chitinized BSF larvae meal diet; IO: BSF larvae oil diet; EX: BSF larvae exoskeleton diet. **Figure S12.** The boxplots for homogeneity of multivariate dispersions in gut microbiota of fish fed experimental diets. CD: control diet; IM: Full-fat BSF (black soldier fly) larvae meal diet; DFIM: Defatted BSF larvae meal diet; DCIM: De-chitinized BSF larvae meal diet; IO: BSF larvae oil diet; EX: BSF larvae exoskeleton diet. **Figure S13.** Number of ASVs mapped to genome-scale metabolic models. Number of samples matched to models at different taxonomic levels (**a**) and the number of models mapped to each sample by taxonomic level (**b**). **Figure S14.** Results from t-tests comparing reaction abundances between pairs of diets. The t-statistic for each reaction is shown along with the mean across all reactions with 95% confidence interval for all significantly enriched subsystems. IO and CD (**a**), EX and IO (**b**), DCIM and CD (**c**), IM and CD (**d**), IO and DFIM (**e**), IO and DCIM (**f**), DFIM and IM (**g**), EX and CD (**h**), EX and DCIM (**i**), IO and IM (**j**), DCIM and IM (**k**), DFIM and CD (**l**), EX and DFIM (**m**), EX and IM (**n**) and DCIM and DFIM (**o**) groups. CD: control diet; IM: full-fat BSF (black soldier fly) larvae meal diet; DFIM: defatted BSF larvae meal diet; DCIM: de-chitinized BSF larvae meal diet; IO: BSF larvae oil diet; EX: BSF larvae exoskeleton diet.

**Additional file 2: Table S1**. Chemical composition (%, as is) of meals and fractions of black soldier fly (BSF) larvae. **Table S2.** Fatty acid composition (% of total fatty acids) of the lipid fraction of black soldier fly larvae. **Table S4**. Pair-wise comparison of alpha diversity indices of gut microbiota in fish fed experimental diets containing meals and fractions of black soldier fly (BSF) larvae – adjusted p values. **Table S5.** PERMANOVA analysis for beta-diversity of gut microbiota in fish fed experimental diets containing meals and fractions of black soldier fly (BSF) larvae – adjusted p value. **Table S6.** Test of homogeneity of multivariate dispersions among dietary groups.

**Additional file 3: Table**. The prevalence of core ASVs in different sample types.

## Data Availability

The raw 16S rRNA gene sequence files and metadata are deposited at the NCBI SRA database under the BioProject PRJNA762510. Other data and code for reproducing the results are available in the GitLab repository (https://gitlab.com/Pabodha/salmon_insects_microbiota_2021).

## Abbreviations

ASVs: Amplicon sequence variants
AMP: Antimicrobial peptides
BSF: Black soldier fly
FA: Fatty acid
GSMMs: Genome-scale metabolic models
LAB: Lactic acid bacteria
LDA: Linear discriminant analysis
LEfSe: Linear discriminant analysis effect size
PD: Phylogenetic diversity
PERMANOVA: Permutation multivariate analysis of variance
PCA: Principal component analysis
PCoA: Principal coordinates analysis
