# Supplementary material for: Modulation of Atlantic salmon (Salmo salar) gut microbiota composition and predicted metabolic capacity by feeding diets with processed black soldier fly (Hermetia illucens) larvae meals and fractions
Source: Anim Microbiome. 2022 Jan 15;4:9. doi: 10.1186/s42523-021-00161-w (PMC8760679; doi:10.1186/s42523-021-00161-w)
Supplement: Supplementary file 1 — Additional file 1: Figure S1. Rarefaction curves based on observed amplicon sequence variants (ASVs) for the different sample types. The ASV table was rarefied based on minimum sequence size (10,332) in the sample for normalization of the sequence for computation of two of the beta-diversity indices (Jaccard distance and unweighted UniFrac distance). CD: control diet; IM: full-fat BSF (black soldier fly) larvae meal diet; DFIM: defatted BSF larvae meal diet; DCIM: de-chitinized BSF larvae meal diet; IO: BSF larvae oil diet; EX: BSF larvae exoskeleton diet. Figure S2. Principal component analysis (PCA) on standardized amplicon sequence variants (ASVs). Score plots for PC1 and PC2 (a) and PC1 and PC3 (b), mean scores (dark) with 95% confidence intervals for PC1 (c), PC2 (d), and PC3 (e), and percentage of variance explained by PCs (f). PC: principal component, CD: control diet; IM: full-fat BSF (black soldier fly) larvae meal diet; DFIM: defatted BSF larvae meal diet; DCIM: de-chitinized BSF larvae meal diet; IO: BSF larvae oil diet; EX: BSF larvae exoskeleton diet. Figure S3. Principal component analysis (PCA) on metabolic reaction abundances (z-scores). Score plots for PC1 and PC2 (a) and PC1 and PC3 (b), mean scores (dark) with 95% confidence intervals for PC1 (c), PC2 (d), and PC3 (e), and percentage of variance explained by PCs (f). PC: principal component, CD: control diet; IM: full-fat BSF (black soldier fly) larvae meal diet; DFIM: defatted BSF larvae meal diet; DCIM: de-chitinized BSF larvae meal diet; IO: BSF larvae oil diet; EX: BSF larvae exoskeleton diet. Figure S4. Expected and observed taxonomic profiles of the mock microbial community standard. Mock_1, Mock_2: observed taxonomic profiles of the mock. Mock_Exp: expected taxonomic profile of the mock. Figure S5. Most abundant taxa in feed samples. Top 10 most abundant taxa at phylum (comprised 100% of abundance) (a) and top 15 most abundant taxa at genus or lowest taxonomy level (comprised 61–75% of abun [file 42523_2021_161_MOESM1_ESM.pdf]

Figure S1

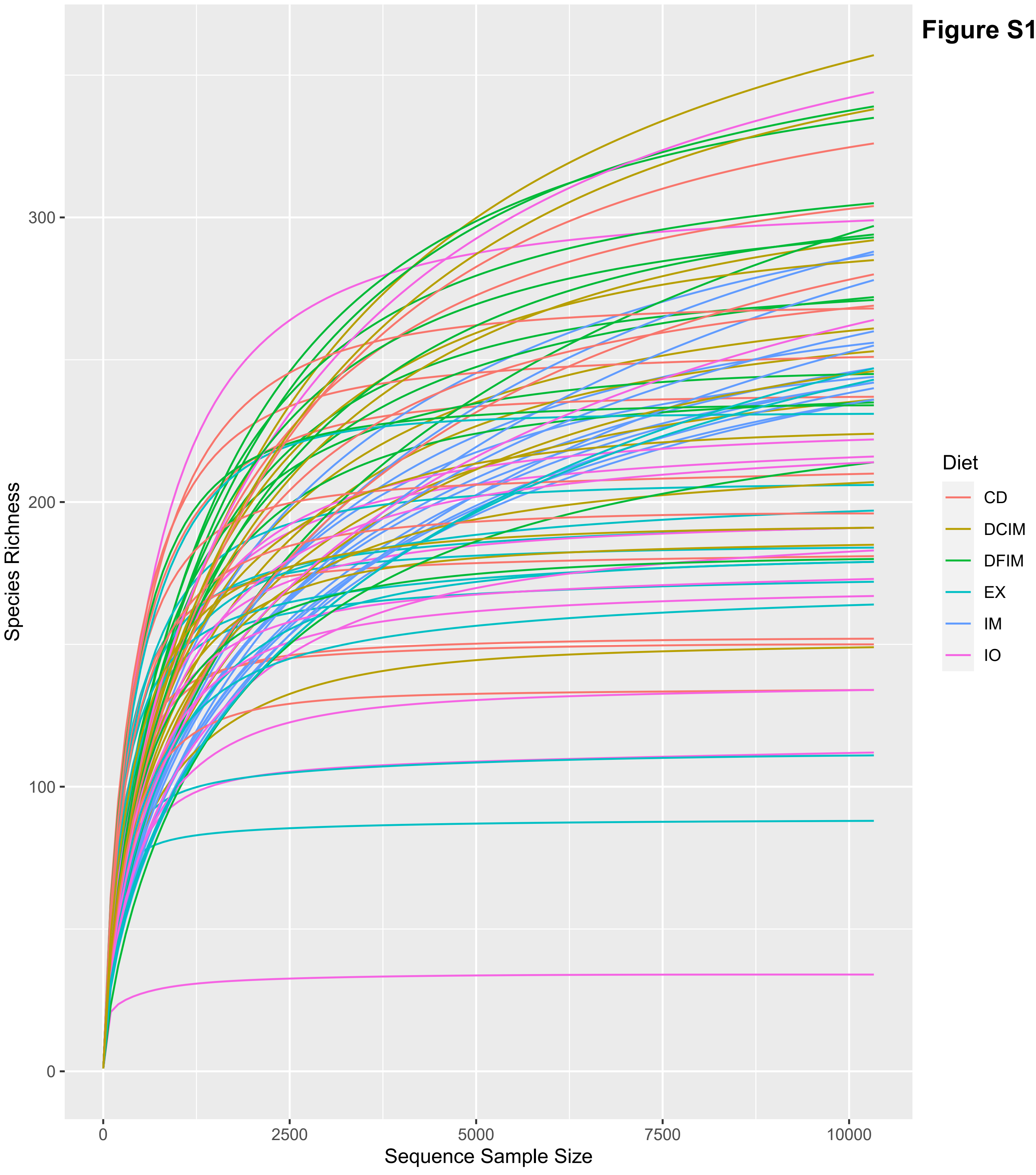

Species Richness
Sequence Sample Size
0
100
200
300
0
2500
5000
7500
10000
Diet
CD
DCIM
DFIM
EX
IM
IO

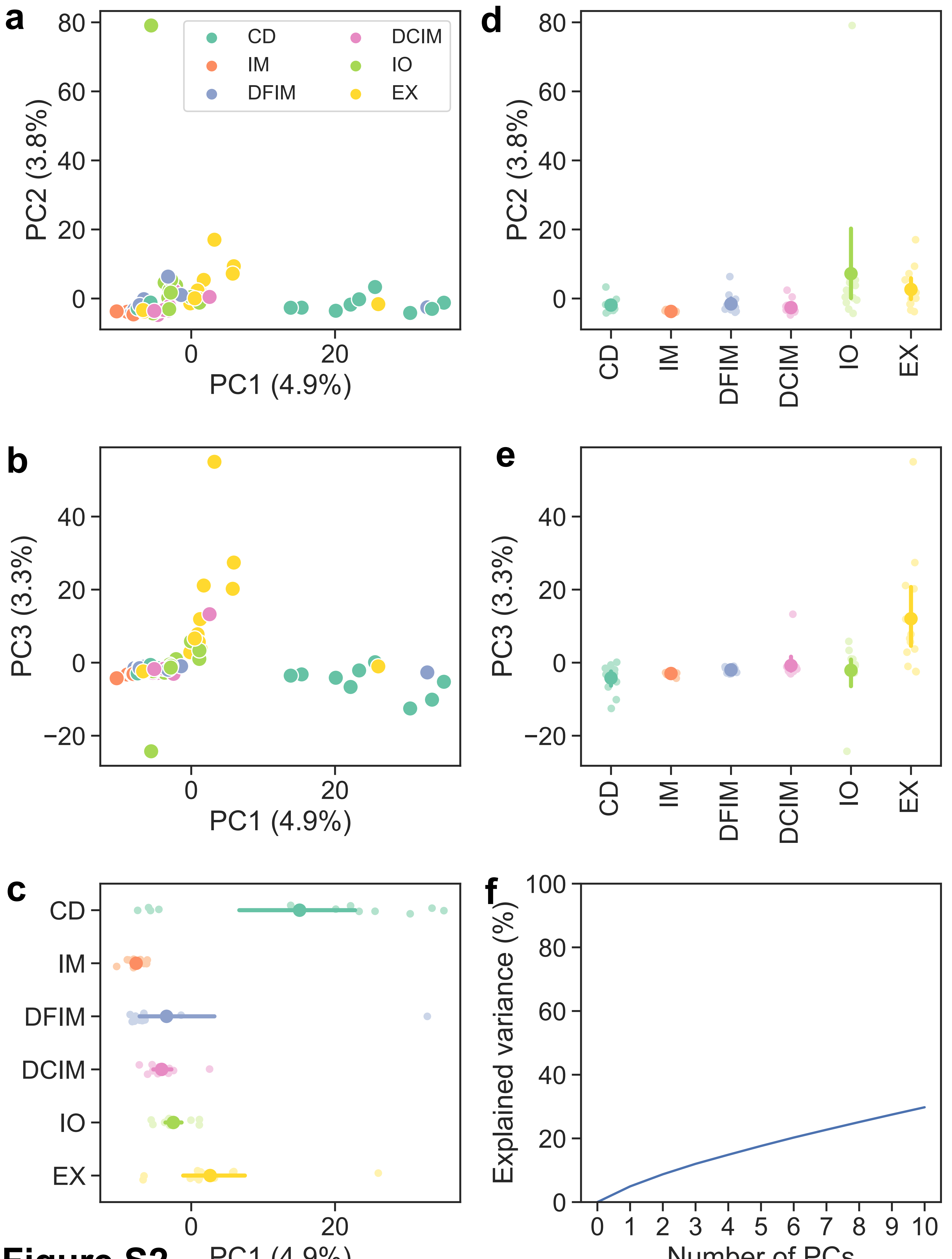

a
b
c
d
e
f
CD
IM
DFIM
DCIM
IO
EX
PC1 (4.9%)
PC2 (3.8%)
PC3 (3.3%)
Explained variance (%)
Number of PCs

Figure S2

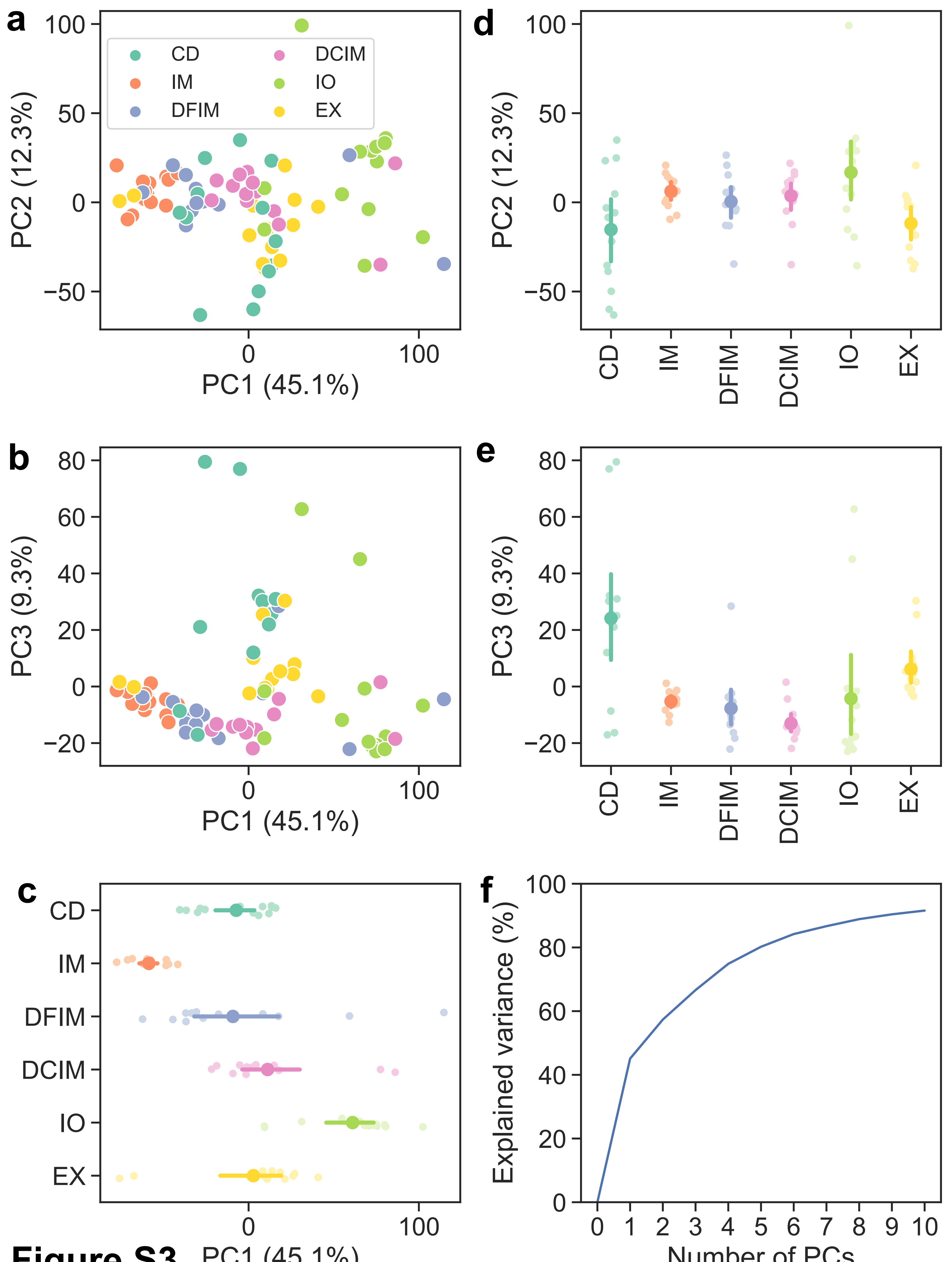
a
b
c
d
e
f
CD
IM
DFIM
DCIM
IO
EX
PC1 (45.1%)
PC2 (12.3%)
PC3 (9.3%)
Explained variance (%)
Number of PCs
100
50
0
−50
80
60
40
20
−20
0 1 2 3 4 5 6 7 8 9 10

Figure S3

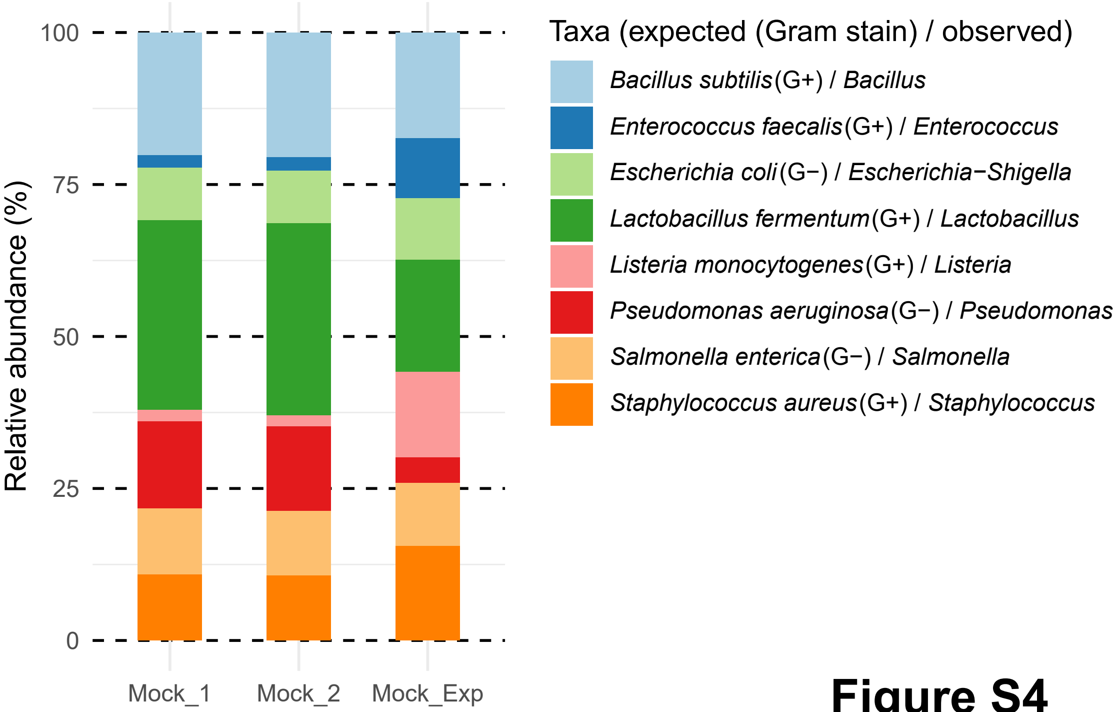

Relative abundance (%)
100
75
50
25
0
Mock_1
Mock_2
Mock_Exp
Taxa (expected (Gram stain) / observed)
Bacillus subtilis(G+) / Bacillus
Enterococcus faecalis(G+) / Enterococcus
Escherichia coli(G−) / Escherichia−Shigella
Lactobacillus fermentum(G+) / Lactobacillus
Listeria monocytogenes(G+) / Listeria
Pseudomonas aeruginosa(G−) / Pseudomonas
Salmonella enterica(G−) / Salmonella
Staphylococcus aureus(G+) / Staphylococcus

Figure S4

Figure S5

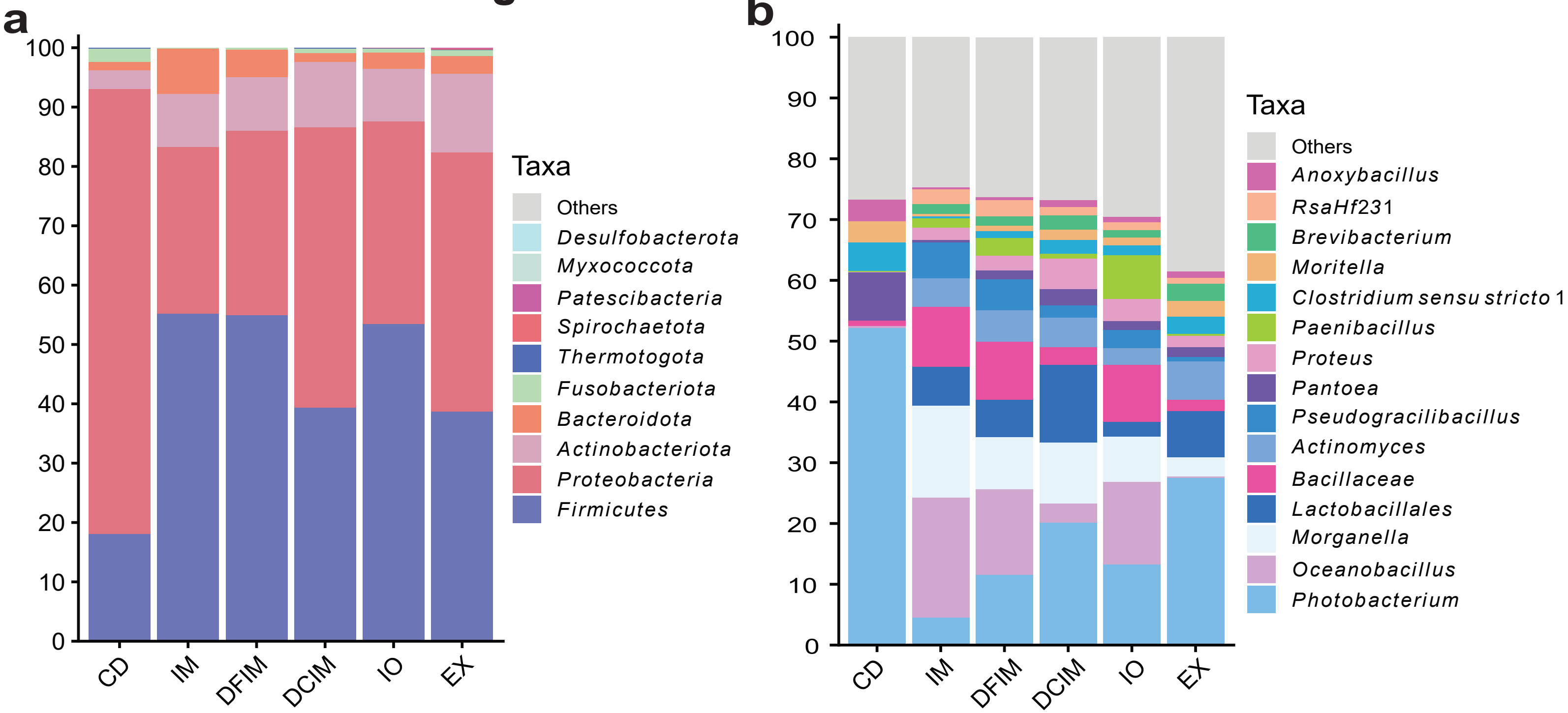
a
b
100
90
80
70
60
50
40
30
20
10
0
CD
IM
DFIM
DCIM
IO
EX
Taxa
Others
Desulfobacterota
Myxococcota
Patescibacteria
Spirochaetota
Thermotogota
Fusobacteriota
Bacteroidota
Actinobacteriota
Proteobacteria
Firmicutes
Taxa
Others
Anoxybacillus
RsaHf231
Brevibacterium
Moritella
Clostridium sensu stricto 1
Paenibacillus
Proteus
Pantoea
Pseudogracilibacillus
Actinomyces
Bacillaceae
Lactobacillales
Morganella
Oceanobacillus
Photobacterium

Figure S6

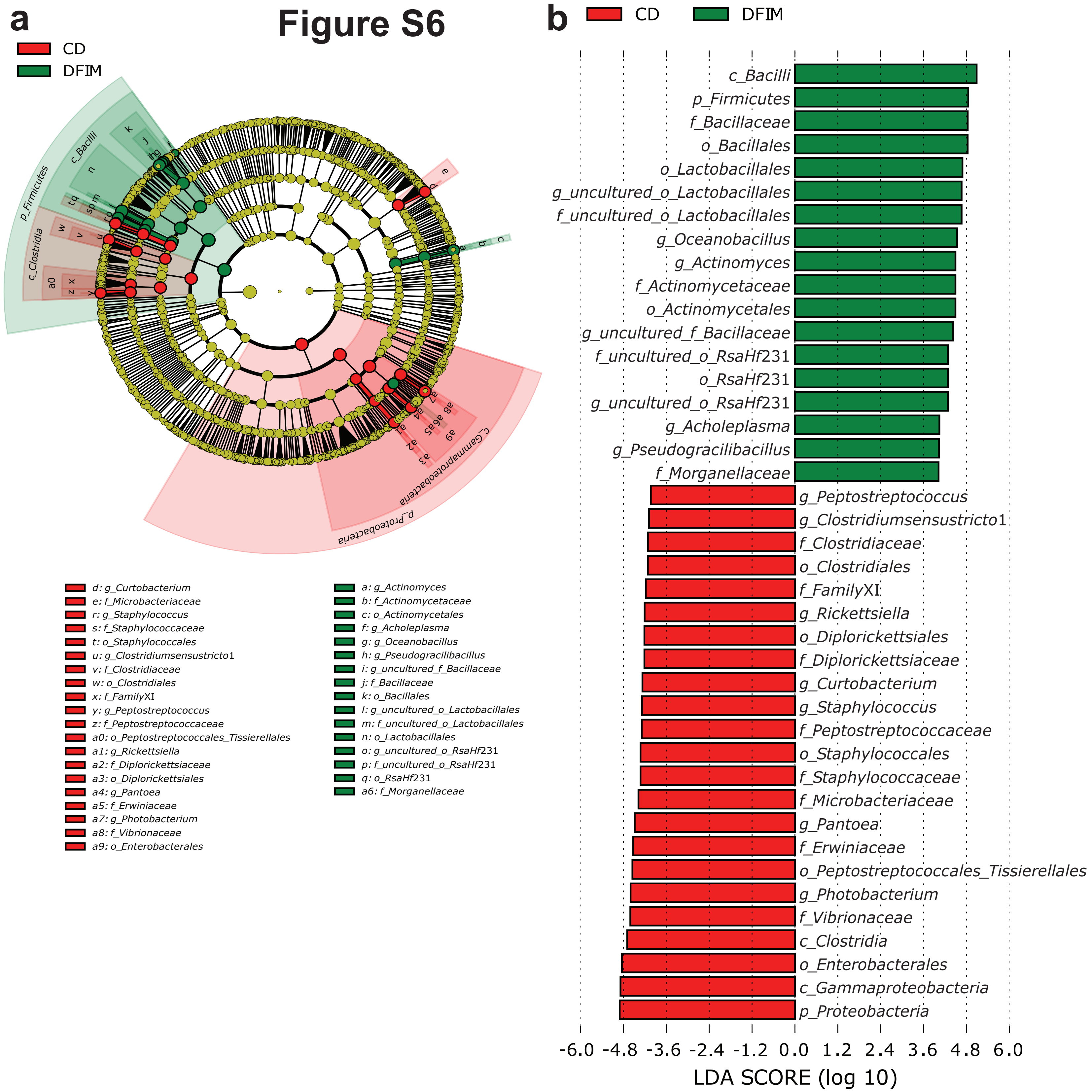
a
CD
DFIM
p_Firmicutes
c_Bacilli
c_Clostridia
c_Gammaproteobacteria
p_Proteobacteria
d: g_Curtobacterium
e: f_Microbacteriaceae
r: g_Staphylococcus
s: f_Staphylococcaceae
t: o_Staphylococcales
u: g_Clostridiumsensustricto1
v: f_Clostridiaceae
w: o_Clostridiales
x: f_FamilyXI
y: g_Peptostreptococcus
z: f_Peptostreptococcaceae
a0: o_Peptostreptococcales_Tissierellales
a1: g_Rickettsiella
a2: f_Diplorickettsiaceae
a3: o_Diplorickettsiales
a4: g_Pantoea
a5: f_Erwiniaceae
a7: g_Photobacterium
a8: f_Vibrionaceae
a9: o_Enterobacterales
a: g_Actinomyces
b: f_Actinomycetaceae
c: o_Actinomycetales
f: g_Acholeplasma
g: g_Oceanobacillus
h: g_Pseudogracilibacillus
i: g_uncultured_f_Bacillaceae
j: f_Bacillaceae
k: o_Bacillales
l: g_uncultured_o_Lactobacillales
m: f_uncultured_o_Lactobacillales
n: o_Lactobacillales
o: g_uncultured_o_RsaHf231
p: f_uncultured_o_RsaHf231
q: o_RsaHf231
a6: f_Morganellaceae
b
CD
DFIM
c_Bacilli
p_Firmicutes
f_Bacillaceae
o_Bacillales
o_Lactobacillales
g_uncultured_o_Lactobacillales
f_uncultured_o_Lactobacillales
g_Oceanobacillus
g_Actinomyces
f_Actinomycetaceae
o_Actinomycetales
g_uncultured_f_Bacillaceae
f_uncultured_o_RsaHf231
o_RsaHf231
g_uncultured_o_RsaHf231
g_Acholeplasma
g_Pseudogracilibacillus
f_Morganellaceae
g_Peptostreptococcus
g_Clostridiumsensustricto1
f_Clostridiaceae
o_Clostridiales
f_FamilyXI
g_Rickettsiella
o_Diplorickettsiales
f_Diplorickettsiaceae
g_Curtobacterium
g_Staphylococcus
f_Peptostreptococcaceae
o_Staphylococcales
f_Staphylococcaceae
f_Microbacteriaceae
g_Pantoea
f_Erwiniaceae
o_Peptostreptococcales_Tissierellales
g_Photobacterium
f_Vibrionaceae
c_Clostridia
o_Enterobacterales
c_Gammaproteobacteria
p_Proteobacteria
-6.0 -4.8 -3.6 -2.4 -1.2 0.0 1.2 2.4 3.6 4.8 6.0
LDA SCORE (log 10)

Figure S7

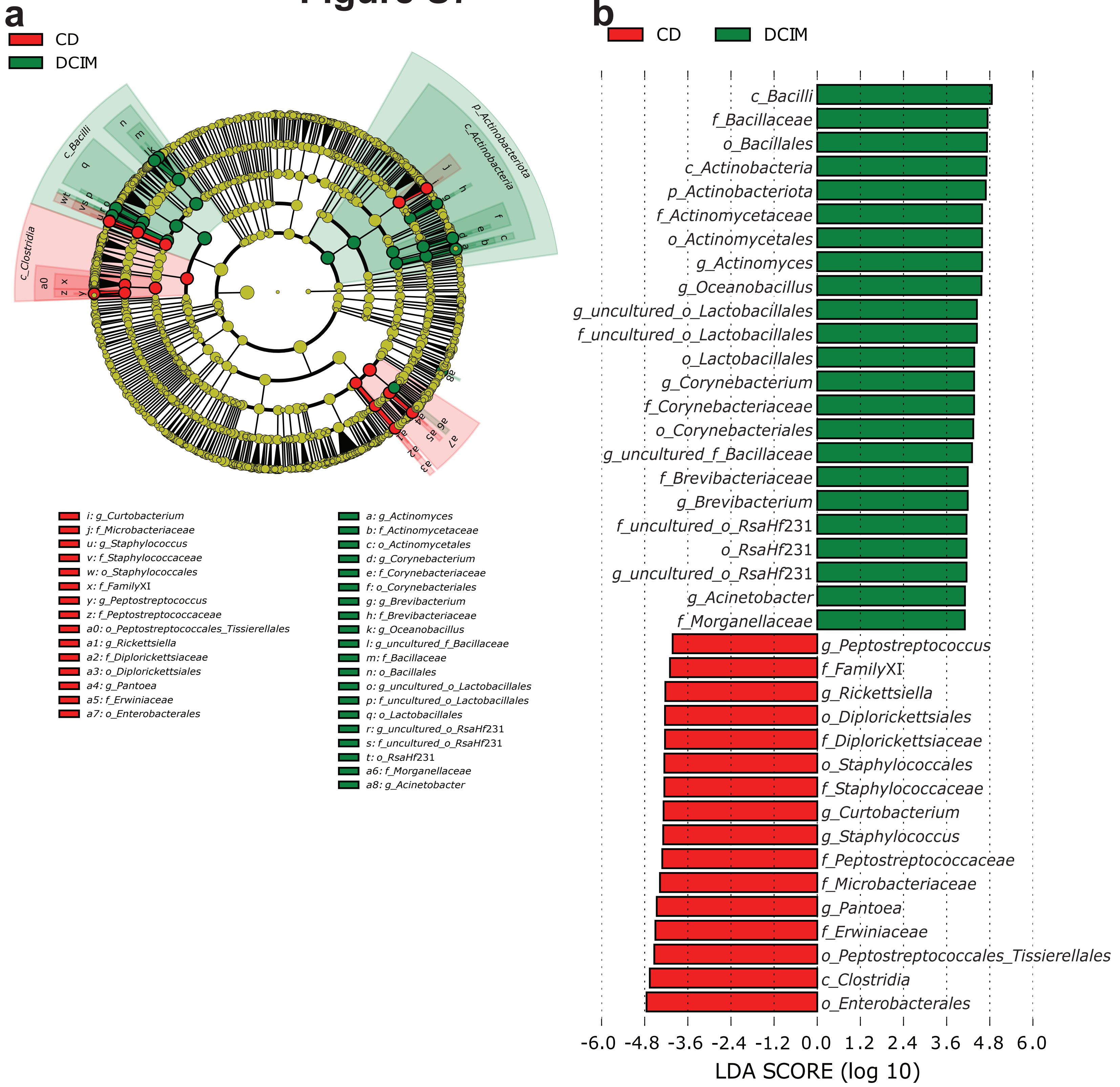
a
CD
DCIM
c_Bacilli
c_Clostridia
p_Actinobacteriota
c_Actinobacteria
i: g_Curtobacterium
j: f_Microbacteriaceae
u: g_Staphylococcus
v: f_Staphylococcaceae
w: o_Staphylococcales
x: f_FamilyXI
y: g_Peptostreptococcus
z: f_Peptostreptococcaceae
a0: o_Peptostreptococcales_Tissierellales
a1: g_Rickettsiella
a2: f_Diplorickettsiaceae
a3: o_Diplorickettsiales
a4: g_Pantoea
a5: f_Erwiniaceae
a7: o_Enterobacterales
a: g_Actinomyces
b: f_Actinomycetaceae
c: o_Actinomycetales
d: g_Corynebacterium
e: f_Corynebacteriaceae
f: o_Corynebacteriales
g: g_Brevibacterium
h: f_Brevibacteriaceae
k: g_Oceanobacillus
l: g_uncultured_f_Bacillaceae
m: f_Bacillaceae
n: o_Bacillales
o: g_uncultured_o_Lactobacillales
p: f_uncultured_o_Lactobacillales
q: o_Lactobacillales
r: g_uncultured_o_RsaHf231
s: f_uncultured_o_RsaHf231
t: o_RsaHf231
a6: f_Morganellaceae
a8: g_Acinetobacter
b
CD
DCIM
c_Bacilli
f_Bacillaceae
o_Bacillales
c_Actinobacteria
p_Actinobacteriota
f_Actinomycetaceae
o_Actinomycetales
g_Actinomyces
g_Oceanobacillus
g_uncultured_o_Lactobacillales
f_uncultured_o_Lactobacillales
o_Lactobacillales
g_Corynebacterium
f_Corynebacteriaceae
o_Corynebacteriales
g_uncultured_f_Bacillaceae
f_Brevibacteriaceae
g_Brevibacterium
f_uncultured_o_RsaHf231
o_RsaHf231
g_uncultured_o_RsaHf231
g_Acinetobacter
f_Morganellaceae
g_Peptostreptococcus
f_FamilyXI
g_Rickettsiella
o_Diplorickettsiales
f_Diplorickettsiaceae
o_Staphylococcales
f_Staphylococcaceae
g_Curtobacterium
g_Staphylococcus
f_Peptostreptococcaceae
f_Microbacteriaceae
g_Pantoea
f_Erwiniaceae
o_Peptostreptococcales_Tissierellales
c_Clostridia
o_Enterobacterales
-6.0 -4.8 -3.6 -2.4 -1.2 0.0 1.2 2.4 3.6 4.8 6.0
LDA SCORE (log 10)

Figure S8

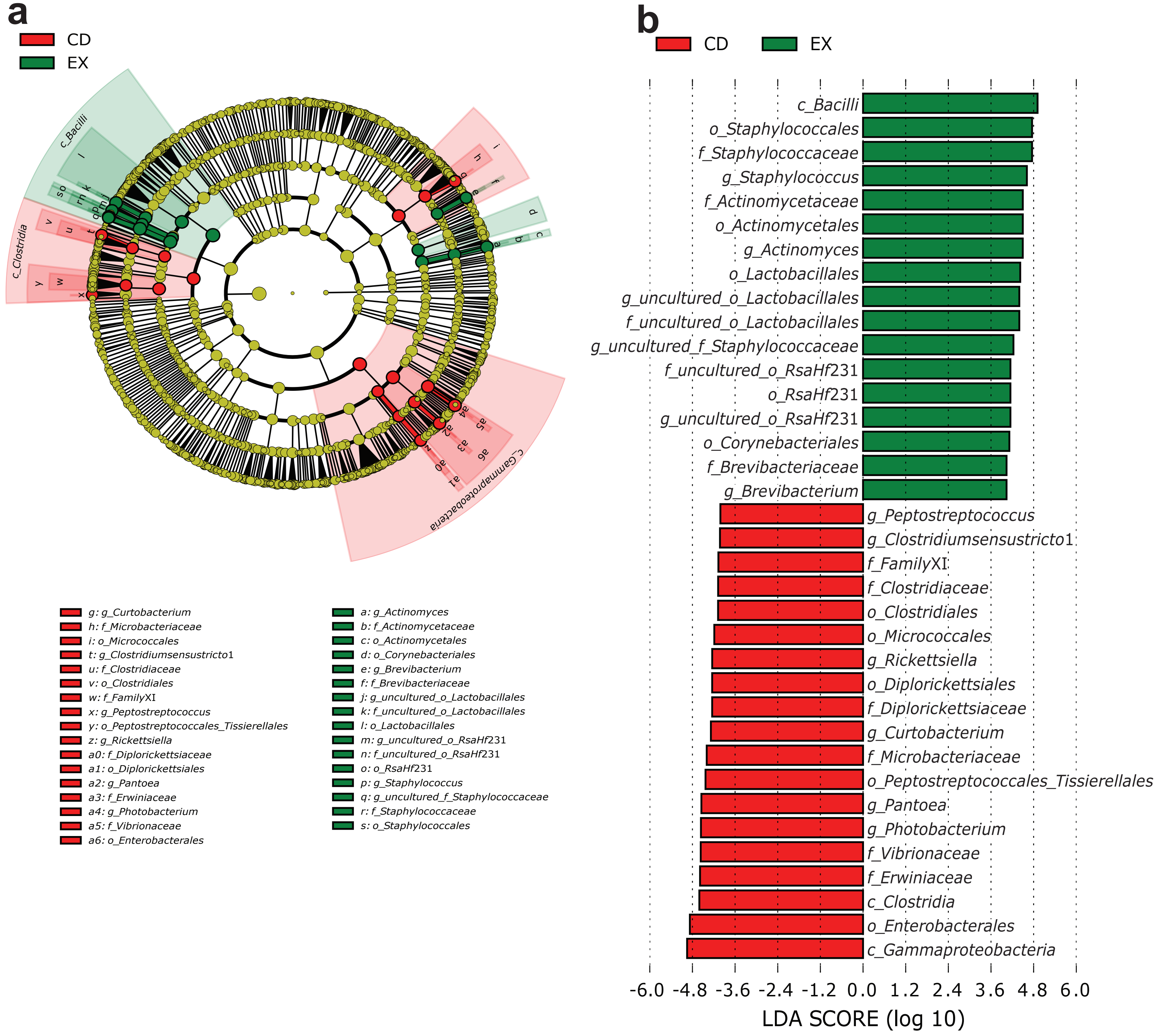
a
CD
EX
c_Bacilli
c_Clostridia
c_Gammaproteobacteria
g: g_Curtobacterium
h: f_Microbacteriaceae
i: o_Micrococcales
t: g_Clostridiumsensustricto1
u: f_Clostridiaceae
v: o_Clostridiales
w: f_FamilyXI
x: g_Peptostreptococcus
y: o_Peptostreptococcales_Tissierellales
z: g_Rickettsiella
a0: f_Diplorickettsiaceae
a1: o_Diplorickettsiales
a2: g_Pantoea
a3: f_Erwiniaceae
a4: g_Photobacterium
a5: f_Vibrionaceae
a6: o_Enterobacterales
a: g_Actinomyces
b: f_Actinomycetaceae
c: o_Actinomycetales
d: o_Corynebacteriales
e: g_Brevibacterium
f: f_Brevibacteriaceae
j: g_uncultured_o_Lactobacillales
k: f_uncultured_o_Lactobacillales
l: o_Lactobacillales
m: g_uncultured_o_RsaHf231
n: f_uncultured_o_RsaHf231
o: o_RsaHf231
p: g_Staphylococcus
q: g_uncultured_f_Staphylococcaceae
r: f_Staphylococcaceae
s: o_Staphylococcales
b
CD
EX
c_Bacilli
o_Staphylococcales
f_Staphylococcaceae
g_Staphylococcus
f_Actinomycetaceae
o_Actinomycetales
g_Actinomyces
o_Lactobacillales
g_uncultured_o_Lactobacillales
f_uncultured_o_Lactobacillales
g_uncultured_f_Staphylococcaceae
f_uncultured_o_RsaHf231
o_RsaHf231
g_uncultured_o_RsaHf231
o_Corynebacteriales
f_Brevibacteriaceae
g_Brevibacterium
g_Peptostreptococcus
g_Clostridiumsensustricto1
f_FamilyXI
f_Clostridiaceae
o_Clostridiales
o_Micrococcales
g_Rickettsiella
o_Diplorickettsiales
f_Diplorickettsiaceae
g_Curtobacterium
f_Microbacteriaceae
o_Peptostreptococcales_Tissierellales
g_Pantoea
g_Photobacterium
f_Vibrionaceae
f_Erwiniaceae
c_Clostridia
o_Enterobacterales
c_Gammaproteobacteria
-6.0 -4.8 -3.6 -2.4 -1.2 0.0 1.2 2.4 3.6 4.8 6.0
LDA SCORE (log 10)

Figure S9

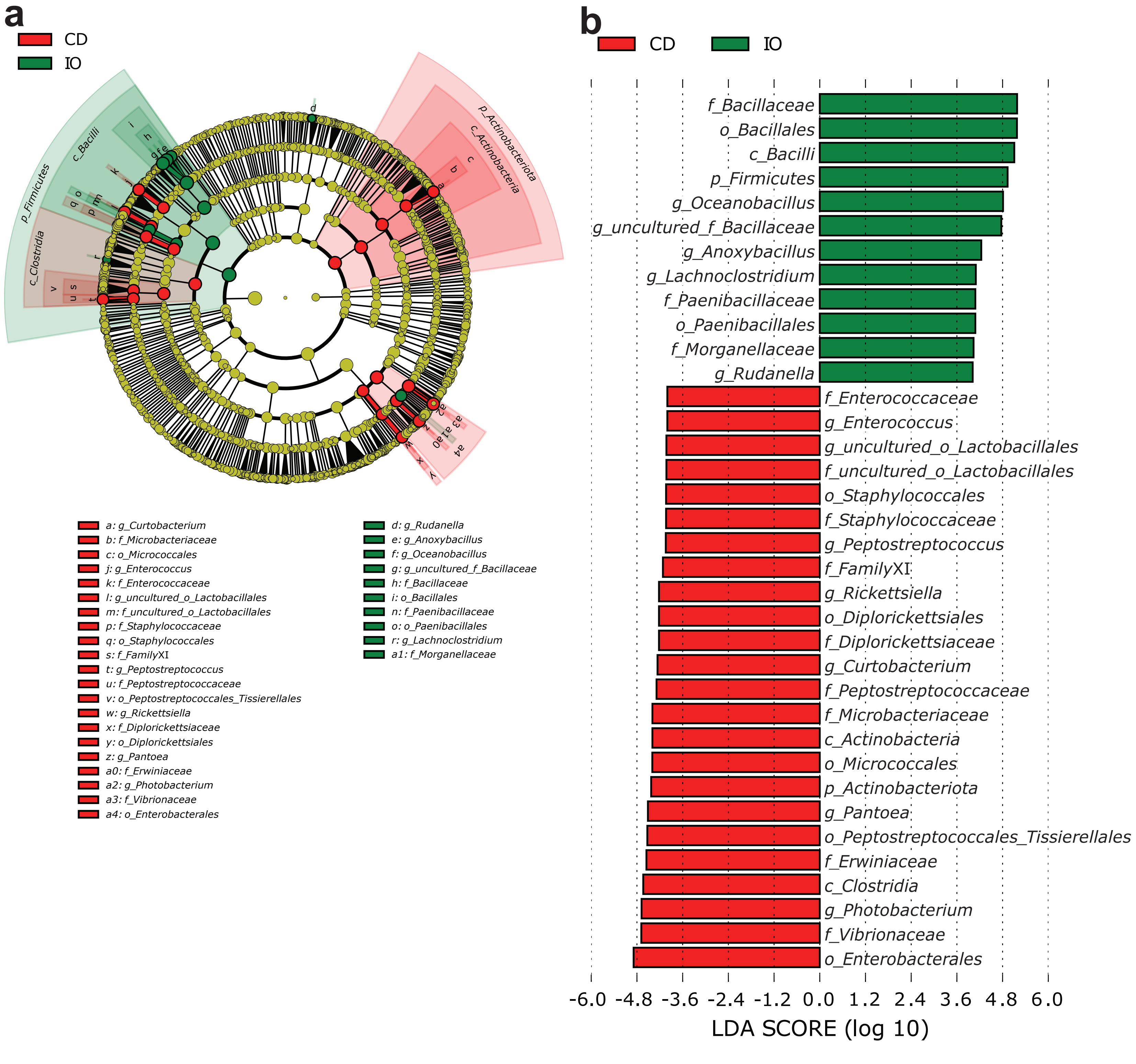

a
CD
IO
p_Firmicutes
c_Bacilli
c_Clostridia
p_Actinobacteriota
c_Actinobacteria
a: g_Curtobacterium
b: f_Microbacteriaceae
c: o_Micrococcales
j: g_Enterococcus
k: f_Enterococcaceae
l: g_uncultured_o_Lactobacillales
m: f_uncultured_o_Lactobacillales
p: f_Staphylococcaceae
q: o_Staphylococcales
s: f_FamilyXI
t: g_Peptostreptococcus
u: f_Peptostreptococcaceae
v: o_Peptostreptococcales_Tissierellales
w: g_Rickettsiella
x: f_Diplorickettsiaceae
y: o_Diplorickettsiales
z: g_Pantoea
a0: f_Erwiniaceae
a2: g_Photobacterium
a3: f_Vibrionaceae
a4: o_Enterobacterales
d: g_Rudanella
e: g_Anoxybacillus
f: g_Oceanobacillus
g: g_uncultured_f_Bacillaceae
h: f_Bacillaceae
i: o_Bacillales
n: f_Paenibacillaceae
o: o_Paenibacillales
r: g_Lachnoclostridium
a1: f_Morganellaceae
b
CD
IO
f_Bacillaceae
o_Bacillales
c_Bacilli
p_Firmicutes
g_Oceanobacillus
g_uncultured_f_Bacillaceae
g_Anoxybacillus
g_Lachnoclostridium
f_Paenibacillaceae
o_Paenibacillales
f_Morganellaceae
g_Rudanella
f_Enterococcaceae
g_Enterococcus
g_uncultured_o_Lactobacillales
f_uncultured_o_Lactobacillales
o_Staphylococcales
f_Staphylococcaceae
g_Peptostreptococcus
f_FamilyXI
g_Rickettsiella
o_Diplorickettsiales
f_Diplorickettsiaceae
g_Curtobacterium
f_Peptostreptococcaceae
f_Microbacteriaceae
c_Actinobacteria
o_Micrococcales
p_Actinobacteriota
g_Pantoea
o_Peptostreptococcales_Tissierellales
f_Erwiniaceae
c_Clostridia
g_Photobacterium
f_Vibrionaceae
o_Enterobacterales
-6.0 -4.8 -3.6 -2.4 -1.2 0.0 1.2 2.4 3.6 4.8 6.0
LDA SCORE (log 10)

Figure S10

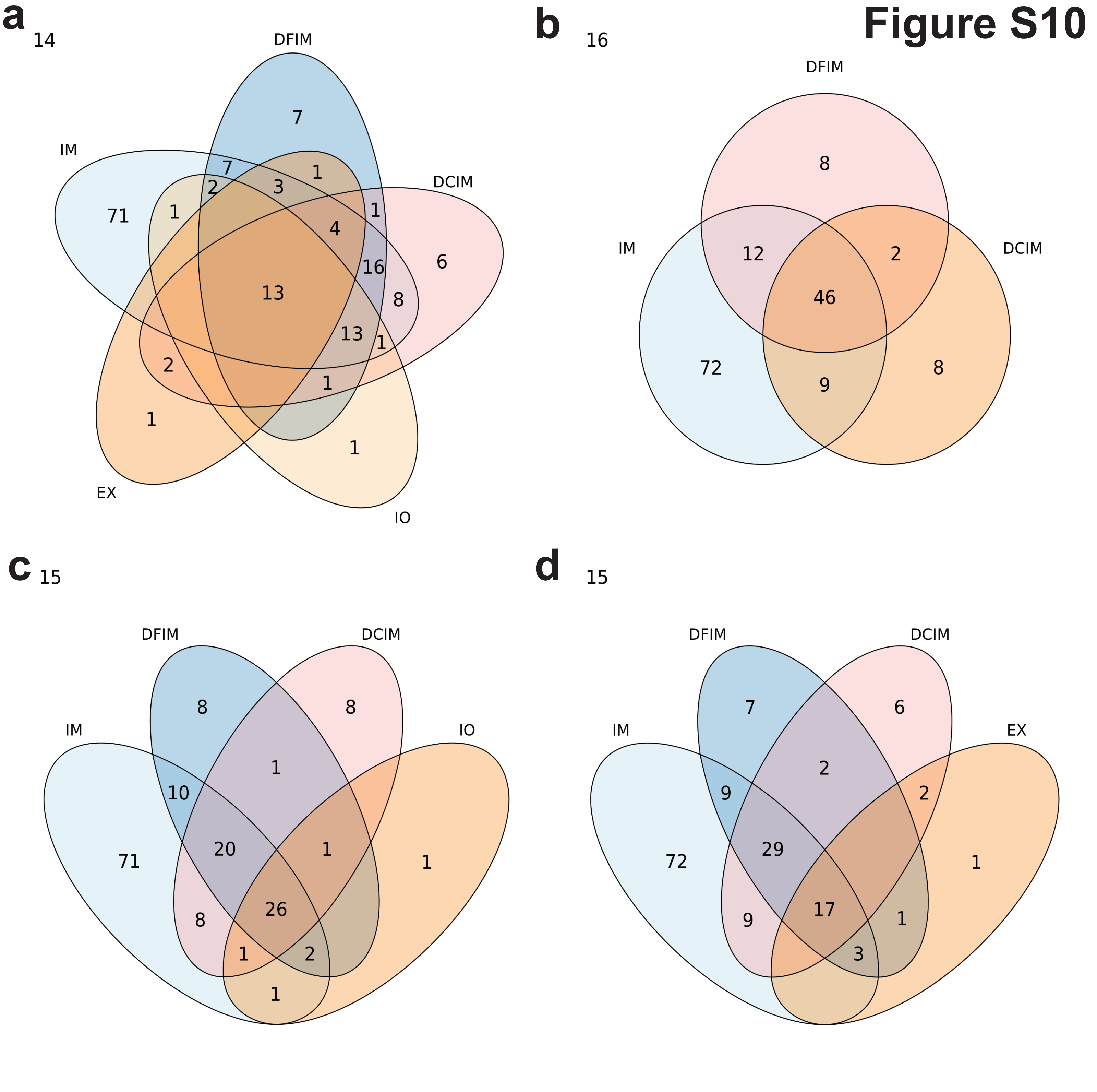
a
14
DFIM
IM
DCIM
EX
IO
7
7
1
2
3
71
1
1
4
16
6
13
8
13
1
2
1
1
1
b
16
DFIM
IM
DCIM
8
12
2
46
72
9
8
c
15
DFIM
DCIM
IM
IO
8
8
1
10
20
1
71
1
26
8
1
2
1
d
15
DFIM
DCIM
IM
EX
7
6
2
9
2
29
72
1
17
9
1
3

Figure S11

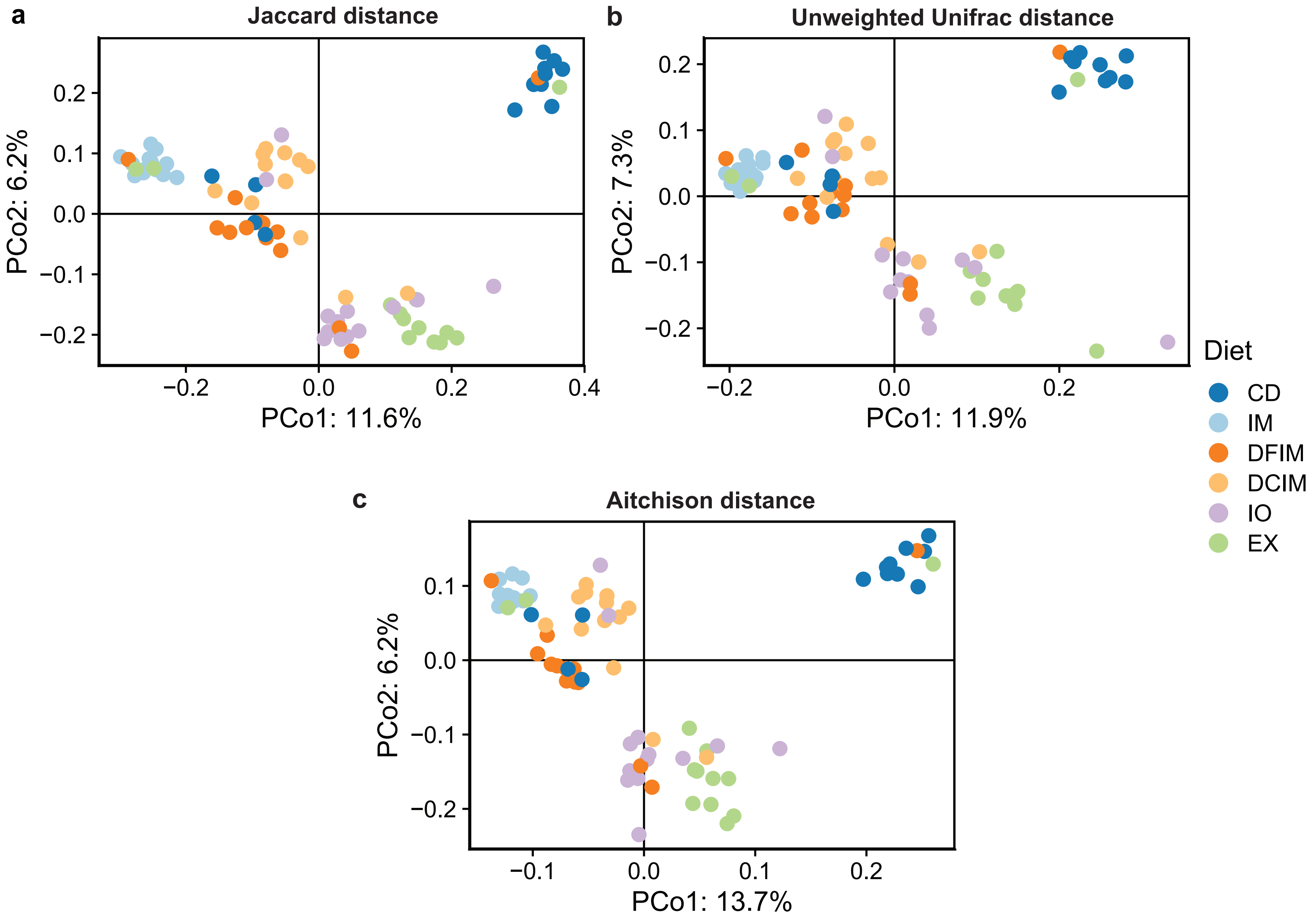

a
Jaccard distance
PCo2: 6.2%
PCo1: 11.6%
0.2
0.1
0.0
−0.1
−0.2
0.0
0.4
b
Unweighted Unifrac distance
PCo2: 7.3%
PCo1: 11.9%
c
Aitchison distance
PCo2: 6.2%
PCo1: 13.7%
−0.1
0.1
Diet
CD
IM
DFIM
DCIM
IO
EX

Figure S12

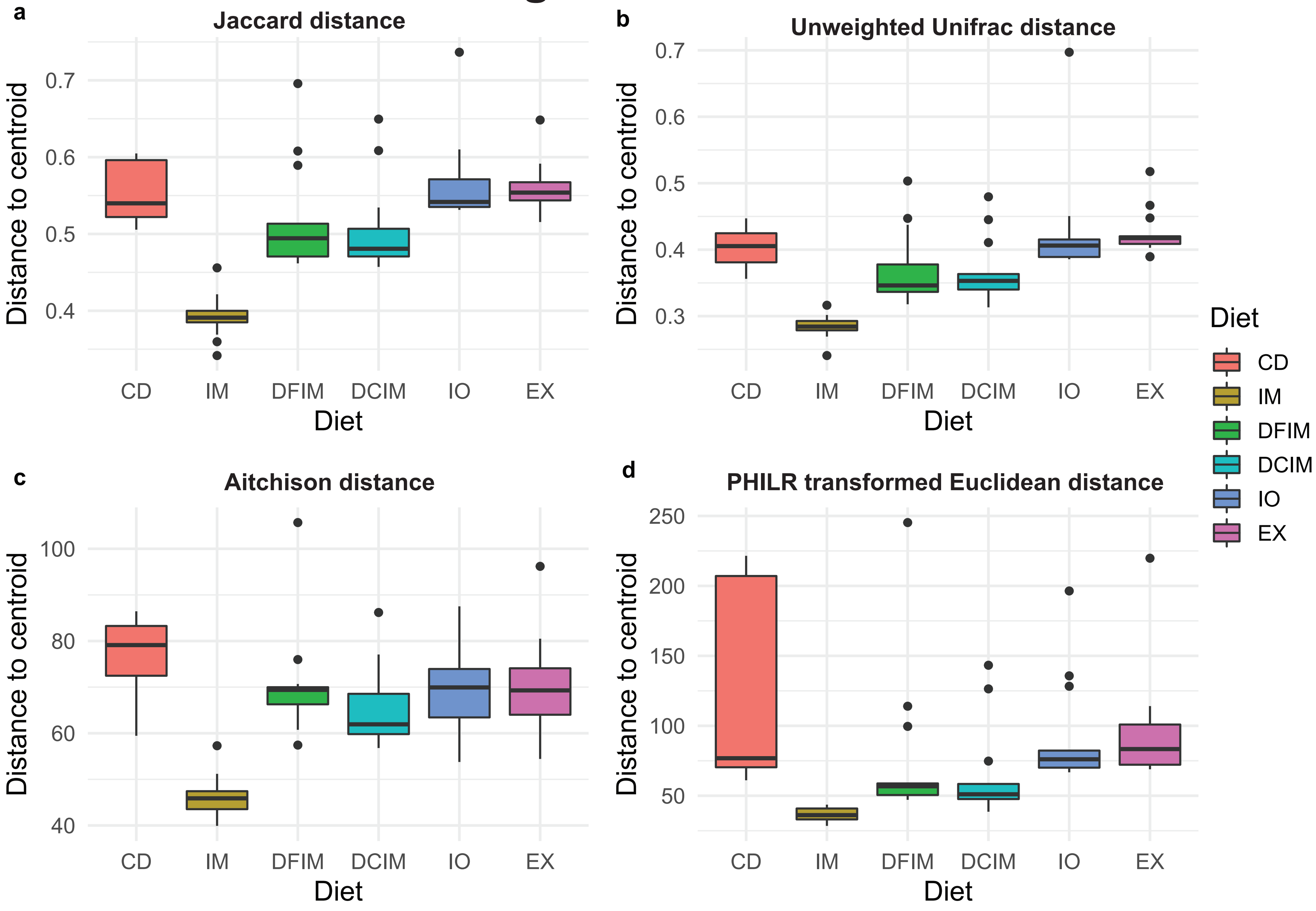
a
Jaccard distance
Distance to centroid
0.4
0.5
0.6
0.7
CD
IM
DFIM
DCIM
IO
EX
Diet
b
Unweighted Unifrac distance
Distance to centroid
0.3
0.4
0.5
0.6
0.7
CD
IM
DFIM
DCIM
IO
EX
Diet
c
Aitchison distance
Distance to centroid
40
60
80
100
CD
IM
DFIM
DCIM
IO
EX
Diet
d
PHILR transformed Euclidean distance
Distance to centroid
50
100
150
200
250
CD
IM
DFIM
DCIM
IO
EX
Diet
Diet
CD
IM
DFIM
DCIM
IO
EX

Figure S13

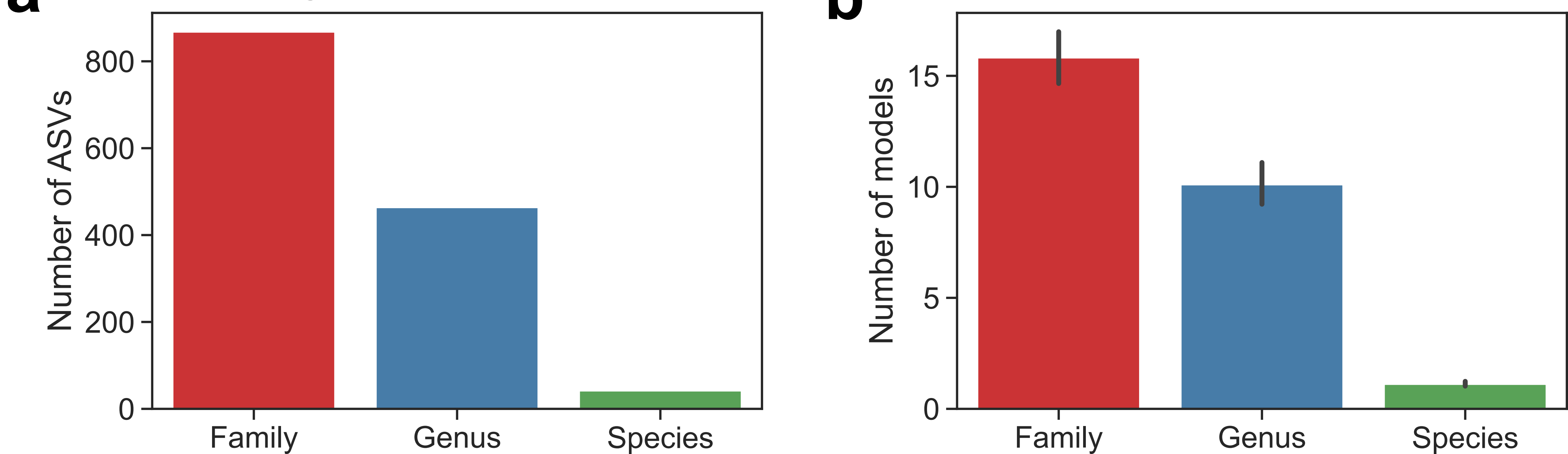
a
Number of ASVs
800
600
400
200
0
Family
Genus
Species
b
Number of models
15
10
5
0
Family
Genus
Species

Figure S14

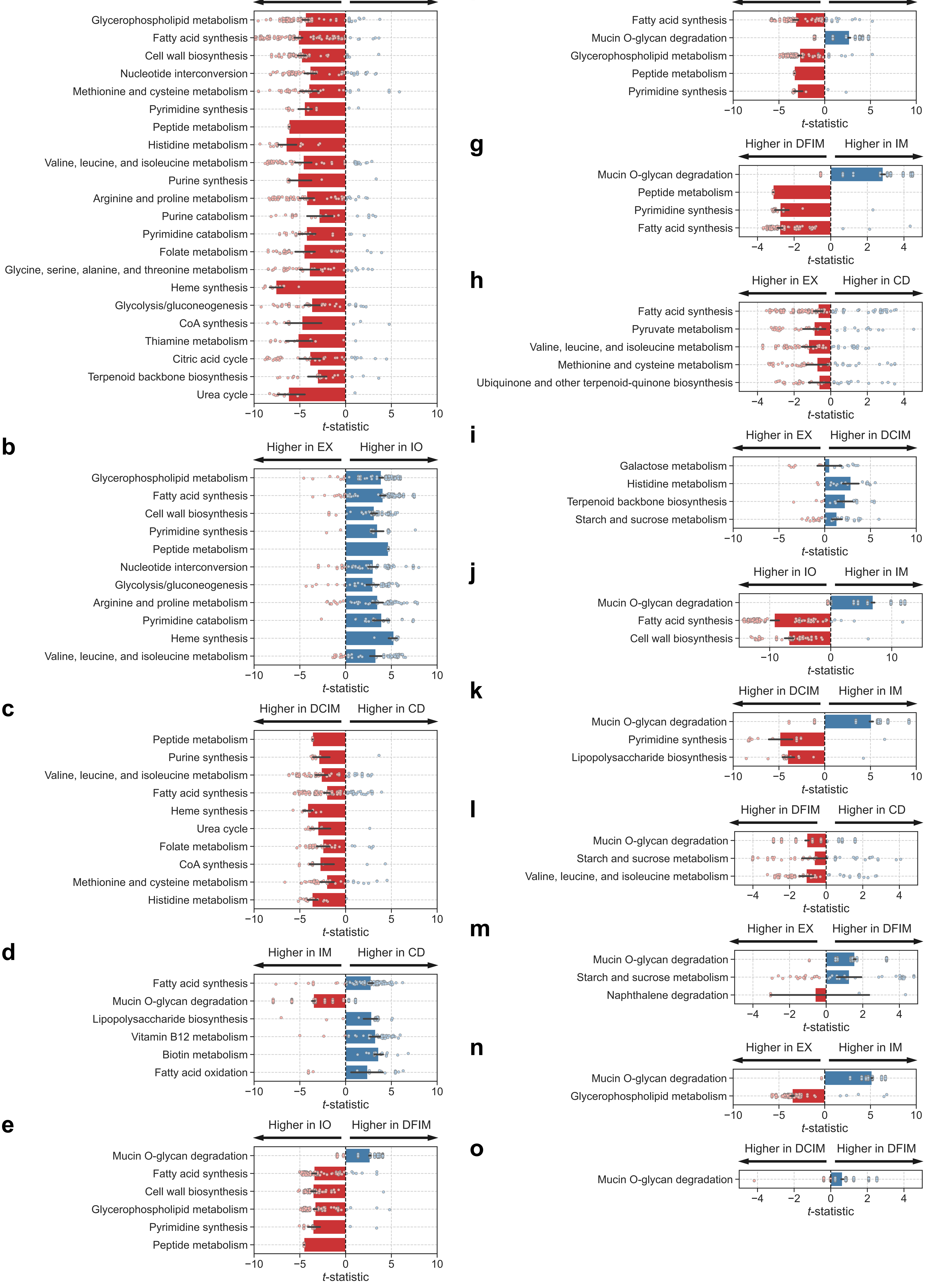
a
Higher in IO
Higher in CD
Glycerophospholipid metabolism
Fatty acid synthesis
Cell wall biosynthesis
Nucleotide interconversion
Methionine and cysteine metabolism
Pyrimidine synthesis
Peptide metabolism
Histidine metabolism
Valine, leucine, and isoleucine metabolism
Purine synthesis
Arginine and proline metabolism
Purine catabolism
Pyrimidine catabolism
Folate metabolism
Glycine, serine, alanine, and threonine metabolism
Heme synthesis
Glycolysis/gluconeogenesis
CoA synthesis
Thiamine metabolism
Citric acid cycle
Terpenoid backbone biosynthesis
Urea cycle
−10 −5 0 5 10
t-statistic
b
Higher in EX
Higher in IO
Glycerophospholipid metabolism
Fatty acid synthesis
Cell wall biosynthesis
Pyrimidine synthesis
Peptide metabolism
Nucleotide interconversion
Glycolysis/gluconeogenesis
Arginine and proline metabolism
Pyrimidine catabolism
Heme synthesis
Valine, leucine, and isoleucine metabolism
−10 −5 0 5 10
t-statistic
c
Higher in DCIM
Higher in CD
Peptide metabolism
Purine synthesis
Valine, leucine, and isoleucine metabolism
Fatty acid synthesis
Heme synthesis
Urea cycle
Folate metabolism
CoA synthesis
Methionine and cysteine metabolism
Histidine metabolism
−10 −5 0 5 10
t-statistic
d
Higher in IM
Higher in CD
Fatty acid synthesis
Mucin O-glycan degradation
Lipopolysaccharide biosynthesis
Vitamin B12 metabolism
Biotin metabolism
Fatty acid oxidation
−10 −5 0 5 10
t-statistic
e
Higher in IO
Higher in DFIM
Mucin O-glycan degradation
Fatty acid synthesis
Cell wall biosynthesis
Glycerophospholipid metabolism
Pyrimidine synthesis
Peptide metabolism
−10 −5 0 5 10
t-statistic
f
Higher in IO
Higher in DCIM
Fatty acid synthesis
Mucin O-glycan degradation
Glycerophospholipid metabolism
Peptide metabolism
Pyrimidine synthesis
−10 −5 0 5 10
t-statistic
g
Higher in DFIM
Higher in IM
Mucin O-glycan degradation
Peptide metabolism
Pyrimidine synthesis
Fatty acid synthesis
−4 −2 0 2 4
t-statistic
h
Higher in EX
Higher in CD
Fatty acid synthesis
Pyruvate metabolism
Valine, leucine, and isoleucine metabolism
Methionine and cysteine metabolism
Ubiquinone and other terpenoid-quinone biosynthesis
−4 −2 0 2 4
t-statistic
i
Higher in EX
Higher in DCIM
Galactose metabolism
Histidine metabolism
Terpenoid backbone biosynthesis
Starch and sucrose metabolism
−10 −5 0 5 10
t-statistic
j
Higher in IO
Higher in IM
Mucin O-glycan degradation
Fatty acid synthesis
Cell wall biosynthesis
−10 0 10
t-statistic
k
Higher in DCIM
Higher in IM
Mucin O-glycan degradation
Pyrimidine synthesis
Lipopolysaccharide biosynthesis
−10 −5 0 5 10
t-statistic
l
Higher in DFIM
Higher in CD
Mucin O-glycan degradation
Starch and sucrose metabolism
Valine, leucine, and isoleucine metabolism
−4 −2 0 2 4
t-statistic
m
Higher in EX
Higher in DFIM
Mucin O-glycan degradation
Starch and sucrose metabolism
Naphthalene degradation
−4 −2 0 2 4
t-statistic
n
Higher in EX
Higher in IM
Mucin O-glycan degradation
Glycerophospholipid metabolism
−10 −5 0 5 10
t-statistic
o
Higher in DCIM
Higher in DFIM
Mucin O-glycan degradation
−4 −2 0 2 4
t-statistic
