# Supplementary material for: Modulation of Atlantic salmon (Salmo salar) gut microbiota composition and predicted metabolic capacity by feeding diets with processed black soldier fly (Hermetia illucens) larvae meals and fractions
Source: Anim Microbiome. 2022 Jan 15;4:9. doi: 10.1186/s42523-021-00161-w (PMC8760679; doi:10.1186/s42523-021-00161-w)
Supplement: Supplementary file 2 — Additional file 2: Table S1. Chemical composition (%, as is) of meals and fractions of black soldier fly (BSF) larvae. Table S2. Fatty acid composition (% of total fatty acids) of the lipid fraction of black soldier fly larvae. Table S4. Pair-wise comparison of alpha diversity indices of gut microbiota in fish fed experimental diets containing meals and fractions of black soldier fly (BSF) larvae – adjusted p values. Table S5. PERMANOVA analysis for beta-diversity of gut microbiota in fish fed experimental diets containing meals and fractions of black soldier fly (BSF) larvae – adjusted p value. Table S6. Test of homogeneity of multivariate dispersions among dietary groups. [file 42523_2021_161_MOESM2_ESM.docx]

**Table S1.** Chemical composition (%, as is) of meals and fractions of black soldier fly (BSF) larvae

| Nutrient | Full-fat BSF larvae meal | Defatted  BSF larvae meal | De-chitinized BSF larvae meal | BSF larvae oil | BSF larvae exoskeleton |
| --- | --- | --- | --- | --- | --- |
| Crude protein | 37.6 | 51.4 | 31.2 | 2 | 59.9 |
| Crude lipid | 29.6 | 11.7 | 43.5 | 96.0 | 11.1 |
| Chitin | 7.05 | 9.65 | 2.15 |  | 19.8 |

**Table S2.** Fatty acid composition (% of total fatty acids) of the lipid fraction of black soldier fly larvae

| Fatty acids | Value |
| --- | --- |
| C8:0 | 0.008 |
| C10:0 | 0.68 |
| C11:0 | 0.015 |
| C12:0 | 39.7 |
| C14:0 | 10.2 |
| C14:1 | 0.21 |
| C15:0 | 0.11 |
| C16:0 | 16.4 |
| C16:1n7 | 2.73 |
| C17:0 | 0.14 |
| C18:0 | 2.93 |
| C18:1n9c | 12.8 |
| C18:2n6c | 12.6 |
| C20:0 | 0.11 |
| C20:1 | 0.085 |
| C18:3n3 | 0.87 |
| C21:0 | 0.28 |
| C20:2n6 | 0.016 |
| C22:0 | 0.017 |
| C20:3n6 | 0.03 |
| C20:3n3 | 0.016 |
| C20:4n6 | 0.007 |
| C24:0 | 0.004 |
| C20:5n3 | 0.006 |
| C24:1 | 0.004 |

**Table S4.** Pair-wise comparison of alpha diversity indices of gut microbiota in fish fed experimental diets containing meals and fractions of black soldier fly (BSF) larvae – adjusted *p* values

| Pair-wise comparisons | Observed ASVs | Pielou’s evenness | Shannon's index | Faith's PD |
| --- | --- | --- | --- | --- |
| IM vs DFIM | 1 | 0.005 | 0.02 | 1 |
| IM vs DCIM | 0.41 | <0.001 | <0.001 | 0.45 |
| IM vs IO | 0.06 | 0.4 | 0.001 | 0.04 |
| IM vs EX | 0.009 | 0.08 | 0.02 | 0.006 |
| DFIM vs DCIM | 1 | 0.84 | 1 | 1 |
| DFIM vs IO | 0.11 | 0.64 | 1 | 0.15 |
| DFIM vs EX | 0.03 | 0.67 | 0.34 | 0.02 |
| DCIM vs IO | 0.91 | 0.64 | 1 | 0.88 |
| DCIM vs EX | 0.26 | 0.64 | 0.34 | 0.29 |
| IO vs EX | 1 | 0.4 | 0.34 | 1 |

IM: Full-fat BSF larvae meal diet; DFIM: Defatted BSF larvae meal diet; DCIM: De-chitinized BSF larvae meal diet; IO: BSF larvae oil diet; EX: BSF larvae exoskeleton diet.

**Table S5.** PERMANOVA analysis for beta-diversity of gut microbiota in fish fed experimental diets containing meals and fractions of black soldier fly (BSF) larvae – adjusted p value

|  | Jaccard distance | Unweighted UniFrac distance | Aitchison distance | Phylogenetic isometric log-ratio (PHILR) transformed Euclidean distance |
| --- | --- | --- | --- | --- |
| F value | 2.84 | 2.94 | 3.01 | 7.64 |
| *p* value | 0.001 | 0.001 | 0.001 | 0.001 |
| Pair-wise comparisons | | | | |
| CD vs IM | 0.02 | 0.02 | 0.02 | 0.02 |
| CD vs DFIM | 0.02 | 0.06 | 0.06 | 0.08 |
| CD vs DCIM | 0.02 | 0.02 | 0.02 | 0.02 |
| CD vs IO | 0.02 | 0.02 | 0.02 | 0.06 |
| CD vs EX | 0.03 | 0.02 | 0.045 | 0.06 |
| IM vs DFIM | 0.02 | 0.02 | 0.02 | 0.02 |
| IM vs DCIM | 0.02 | 0.02 | 0.02 | 0.02 |
| IM vs IO | 0.02 | 0.02 | 0.02 | 0.02 |
| IM vs EX | 0.02 | 0.02 | 0.02 | 0.02 |
| DFIM vs DCIM | 0.03 | 0.02 | 0.02 | 0.18 |
| DFIM vs IO | 0.03 | 0.03 | 0.03 | 1 |
| DFIM vs EX | 0.06 | 0.02 | 0.02 | 0.02 |
| DCIM vs IO | 0.02 | 0.045 | 0.09 | 0.12 |
| DCIM vs EX | 0.02 | 0.03 | 0.02 | 0.02 |
| IO vs EX | 0.06 | 0.08 | 0.02 | 0.02 |

CD: Control diet; IM: Full-fat BSF larvae meal diet; DFIM: Defatted BSF larvae meal diet; DCIM: De-chitinized BSF larvae meal diet; IO: BSF larvae oil diet; EX: BSF larvae exoskeleton diet.

**Table S6.** Test of homogeneity of multivariate dispersions among dietary groups

|  | Jaccard distance | Unweighted UniFrac distance | Aitchison distance | Phylogenetic isometric log-ratio (PHILR) transformed Euclidean distance |
| --- | --- | --- | --- | --- |
| F value | 22.2 | 15.5 | 17.4 | 5.14 |
| *p* value | 0.001 | 0.001 | 0.001 | 0.002 |
| Pair-wise comparisons | | | | |
| CD vs IM | 0.001 | 0.001 | 0.001 | 0.001 |
| CD vs DFIM | 0.13 | 0.09 | 0.15 | 0.11 |
| CD vs DCIM | 0.02 | 0.03 | 0.002 | 0.02 |
| CD vs IO | 0.51 | 0.32 | 0.04 | 0.29 |
| CD vs EX | 0.65 | 0.11 | 0.13 | 0.38 |
| IM vs DFIM | 0.001 | 0.001 | 0.001 | 0.004 |
| IM vs DCIM | 0.001 | 0.001 | 0.001 | 0.007 |
| IM vs IO | 0.001 | 0.001 | 0.001 | 0.001 |
| IM vs EX | 0.001 | 0.001 | 0.001 | 0.001 |
| DFIM vs DCIM | 0.59 | 0.88 | 0.20 | 0.52 |
| DFIM vs IO | 0.07 | 0.049 | 0.76 | 0.43 |
| DFIM vs EX | 0.07 | 0.007 | 0.97 | 0.31 |
| DCIM vs IO | 0.01 | 0.042 | 0.23 | 0.07 |
| DCIM vs EX | 0.006 | 0.003 | 1.91 | 0.03 |
| IO vs EX | 0.77 | 0.86 | 0.80 | 0.76 |

CD: Control diet; IM: Full-fat BSF larvae meal diet; DFIM: Defatted BSF larvae meal diet; DCIM: De-chitinized BSF larvae meal diet; IO: BSF larvae oil diet; EX: BSF larvae exoskeleton diet.
